# Predicting upcoming visual features during eye movements yields scene representations aligned with human visual cortex

**DOI:** 10.1126/sciadv.aee1955

**Published:** 2026-10-21

**Authors:** Sushrut Thorat, Adrien Doerig, Alexander Kroner, Carmen Amme, Tim C. Kietzmann

**Affiliations:** ^1^ Institute of Cognitive Science, Osnabrück University, Osnabrück 49069, Germany.; ^2^ Lossfunk, Bengaluru 560038, India.; ^3^ Department of Education and Psychology, Freie Universität, Berlin 14195, Germany.; ^4^ Bernstein Center for Computational Neuroscience, Berlin 10115, Germany.

## Abstract

Natural scenes are complex arrangements of objects, surfaces, and backgrounds. For the brain’s visual system to effectively operate, it needs to extract not only what objects are present but also their spatial and semantic relations. We hypothesize that such structures may be learned, in a self-supervised fashion, by exploiting temporal regularities of natural active vision: Each fixation reveals a glimpse that is related to the previous one via co-occurrence and saccade-conditioned spatial regularities. We instantiate this idea with glimpse prediction networks (GPNs), recurrent models trained to predict the embedding of the next glimpse along human-like scanpaths. GPNs are shown to successfully extract complex scene information, including object co-occurrences and spatial object arrangements, and integrate information across glimpses. GPN representations align strongly with human fMRI responses in mid- and higher-level visual cortex and match, often outperform, alternative state-of-the-art ANN models, establishing next-glimpse-prediction as a biologically plausible route toward brain-aligned scene representations.

## INTRODUCTION

The advent of neuroconnectionism in the past decade delivered image-computable models of visual-cortex representations of natural object images (*1*, *2*). While most prior success was reported for representations of single (natural) objects (*3*), natural scenes pose a tougher challenge. Scenes contain multiple objects, occlusions, surfaces, and backgrounds, all of which are in spatial and semantic relations with each other (*4*, *5*). What approach could yield neural networks that are able to unify these scene parts and their relations into brain-aligned scene representations?

As standard object-classification objectives do not explicitly supervise such relations, in search for an alternative, recent studies turned to large-language model (LLM) embeddings of scene captions. These embeddings contain information of namable scene parts and their relations, which were used as a supervised learning target. Networks trained with this objective acquired representations that aligned better with scene representations across visual cortex than object-classification networks (*6*, *7*). Yet, two issues with this approach remain. First, the LLM embeddings are themselves based on large amounts of training data, raising questions about their biological plausibility (*8*). Second, LLM embeddings were also shown to align with scene representations of macaques, which do not have access to complex language (*9*). These observations raise the question of how such rich, brain-aligned scene representations could be learned without language.

We propose that an answer lies in the information available to the brain during active vision. Humans explore the world through sequences of fixations, with each fixation delivering a high-resolution glimpse of a part of the visual scene in front of us (*10*). Sequences of glimpses are rich sources of information. For example, they encode co-occurrence statistics of scene parts (e.g., tiles → sink is likely; tiles → whale is not) (*11*) as well as information about their spatial arrangement, conveyed by saccadic efference copies (e.g., from a car, an upward eye movement tends to reveal sky, not road) (*12*). To induce these statistics into neural networks, we take inspiration from computational work on structure discovery in sequences (*13*): We propose that predicting the visual features of the upcoming glimpse could serve as a meaningful objective as it requires knowing what scene parts could occur next given the saccade. Additionally, recurrence could help build a unified scene representation: Executing sequential predictions may benefit from the ability to integrate information over glimpses to improve future predictions. Following this logic, we hypothesized that predicting the next glimpse features with a recurrent architecture may constitute an objective that would lead to rich, unified, scene representations.

To test this hypothesis, we designed glimpse prediction networks (GPNs): recurrent deep neural networks trained to predict the post-saccadic features, operationalized as high-level embeddings of glimpse pixels from pretrained networks, in human-like glimpse sequences over natural-scene images (Fig. 1A). Model variants include GPNs with/without recurrence and with/without saccadic information. We report that GPNs are able to acquire knowledge of feature co-occurrence and, when saccade information is made available, spatial arrangement of natural scene parts. With recurrence, they can infer and integrate such relationships across parts in novel scenes. The GPN parameters were trained entirely through self-supervised next-glimpse prediction, without scene labels, language supervision, or neural data; the visual backbones and DeepGaze III sampling model were pretrained and frozen. The resulting internal representations were found to align well with scene representations in human visual cortex, better than data/parameter-matched networks supervised to predict caption embeddings, and state-of-the-art vision and vision-language neural networks. These findings highlight a biologically meaningful path toward self-supervised models of human scene representation.

**Fig. 1.**
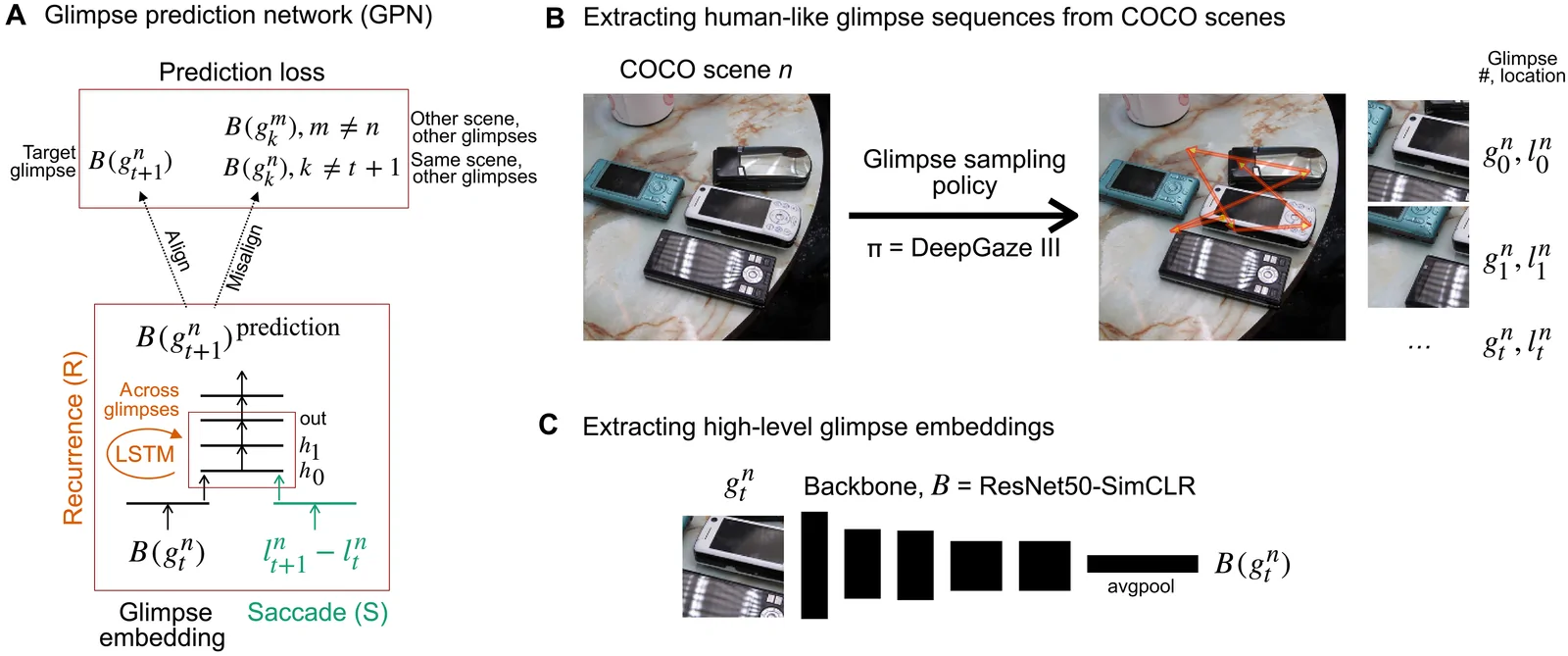
GPNs for scene understanding. (**A**) GPNs took glimpse embeddings and predicted the next glimpse embeddings in a sequence, with optional access to saccades (S) and recurrence (R). They were trained with a contrastive prediction loss. We trained the four GPN variants—GPN-B (no saccades and no recurrence), GPN-R, GPN-S, and GPN-RS (saccades and recurrence)—and assessed their behavior and the alignment of their internal representations with scene representations in the human visual cortex. (**B**) The glimpse centers were sampled from human-like eye-movement sequences over 256-px COCO scenes, modeled with DeepGaze III. Glimpses (91 px) were cropped around these centers. (**C**) The glimpse embeddings were extracted with pretrained backbones to encode high-level visual features. For example, the primary results were obtained using the ResNet50-SimCLR backbone, which is a ResNet50 trained with the SimCLR self-supervised learning objective on the ILSVRC dataset. The avgpool representation of the glimpse was used as its embedding. These backbones were frozen during GPN training. Photo Credits: [(B) and (C)] “4 Students 4 Phones,” Alan Levine, Flickr, CC0 1.0. Images were cropped and annotated from the originals.

## RESULTS

GPNs are trained with a contrastive objective (*14*, *15*) to predict the (high-level) embedding of the next saccade target (glimpse), given a sequence of prior fixations and saccadic efferent copies (interglimpse displacements). That is, the generated prediction should be closer (in cosine similarity terms) to the embedding of the next glimpse and farther from the embeddings of other glimpses from the same scene or other scenes. To better understand the impact of the various ingredients, GPN variants were created using a 2 × 2 design with the factors recurrence (presence denoted as R, implemented as an LSTM or its nonrecurrent equivalent) and saccade target location (presence denoted with S), implemented as a two-dimensional (2D) displacement vector from the center of the given glimpse to the center of the next glimpse (Fig. 1A). The model variant with neither recurrence nor saccadic efferent copies was denoted B (baseline). Human-like input glimpse sequences were modeled using DeepGaze III (*16*), over Common Objects in Context (COCO) scene images (Fig. 1B) (*17*). We used DeepGaze III because it predicts scanpaths, not just static saliency: Given an image and fixation history, it predicts a distribution over the next fixation location, from which human-like glimpse sequences can be sampled. It also reported state-of-the-art performance on free-viewing scanpath prediction at the time. Reliance of the results reported below (specifically, the neural-alignment results) on the components of this glimpse-sampling policy is depicted in fig. S1. To obtain a high-level embedding of the glimpses, we used a self-supervised, SimCLR-trained ResNet50 (Fig. 1C), but note that similar results can be obtained with other supervised and self-supervised backbones (RN50-ILSVRC and DINOv2-B; see figs. S2 and S3).

### GPNs learn the spatial co-occurrence of scene parts and integrate across them

We first asked whether next-glimpse prediction produces three key properties of scene representations: sensitivity to which scene parts co-occur, sensitivity to their spatial arrangement, and integration of information across successive glimpses. We qualitatively assessed the behavior of GPN-RS, the variant with the lowest prediction loss, on held-out COCO scenes (“test-515”). As seen in Fig. 2A (examples across variants in fig. S2), GPN-RS predictions reflected both scene-level co-occurrence and the queried saccadic displacement. In one scene, an input embedding of a glimpse containing the rear of a car, paired with a saccade toward the upper right, led the model to predict an embedding whose nearest neighbor corresponded to an upper/front portion of the same car. Later, in the same sequence, querying the model from $g_{2}^{393}$ with an upward saccade led to a nearest-neighbor prediction containing blue sky. In another scene, saccades moving away from an initial grass glimpse led to predictions aligned with cattle-containing glimpses, consistent with learned scene co-occurrence. After the model received a subsequent glimpse containing part of a child, its later predictions shifted accordingly: nearest-neighbor matches contained child-related content and their spatial arrangement followed the queried saccades. These examples suggest that GPN-RS predictions were not simple nearest-neighbor matches to the input glimpse, but reflected saccade-conditioned scene expectations updated by the accumulated glimpse sequence.

**Fig. 2.**
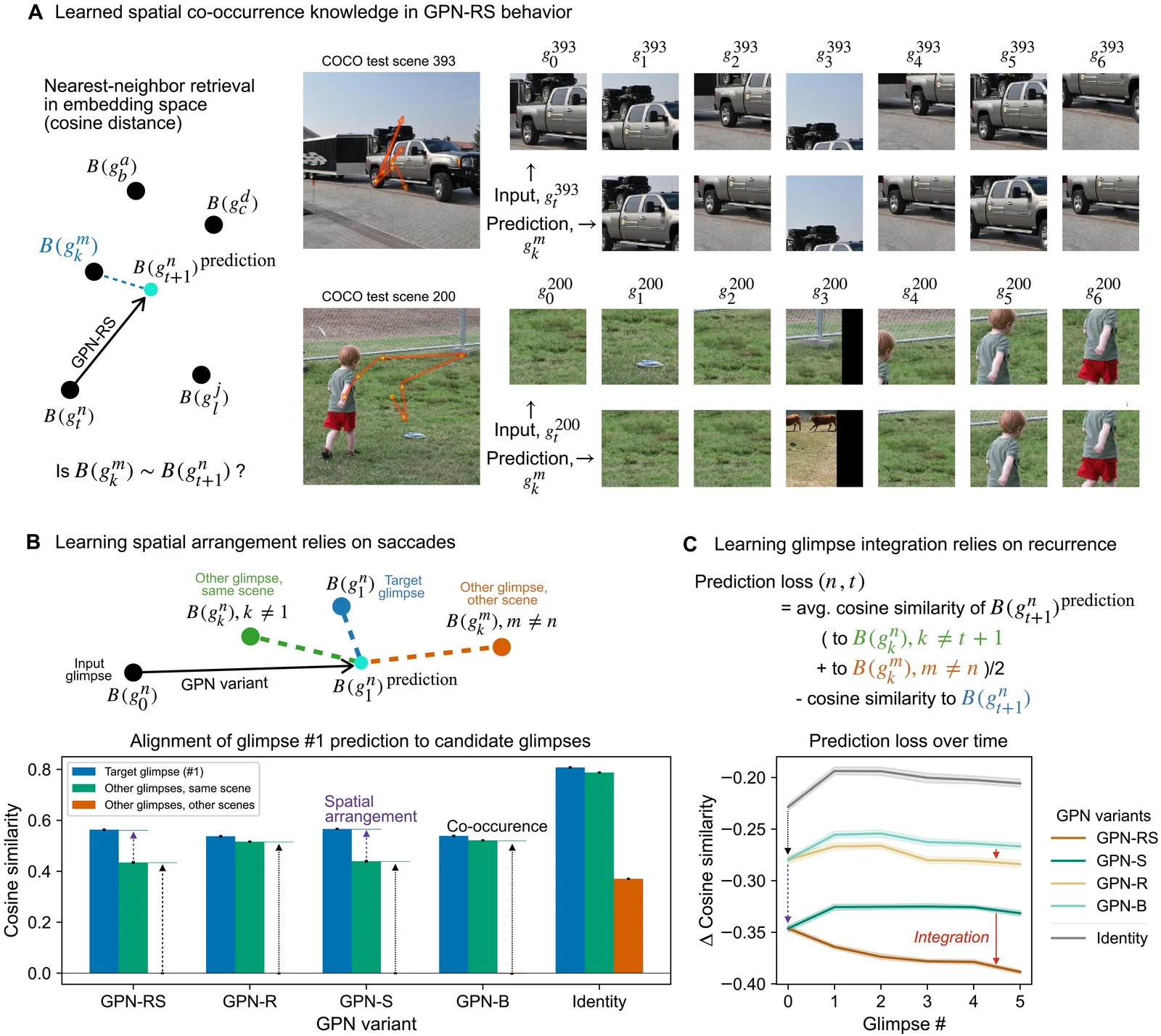
GPNs learn co-occurrence, spatial arrangement, and integration of scene parts. (**A**) Qualitative examples of GPN-RS predictions on held-out COCO scenes. Given an input glimpse embedding and a queried saccade, GPN-RS predicted an embedding whose nearest neighbor often corresponded to scene content consistent with both the current glimpse sequence and the requested spatial displacement. In one scene, a saccade from a rear-car glimpse toward the upper right led to predictions aligned with an upper/front-car glimpse; a later upward saccade led to predictions aligned with sky-containing glimpses. In another scene, saccades from a grass glimpse led to predictions aligned with cattle-containing glimpses, and later predictions incorporated newly observed child-related content while preserving the queried spatial relations. Nearest-neighbor retrievals were computed in backbone embedding space using cosine distance. (**B**) GPN predictions captured co-occurrence and spatial-arrangement statistics. Predictions from the first, central, glimpse ($g_{0}$) were compared with the target next glimpse, other glimpses from the same scene, and glimpses from other scenes. All GPNs’ predictions aligned more strongly with same-scene glimpses than with other-scene glimpses, indicating learned co-occurrence structure. GPN-S and GPN-RS, which had access to saccades, had predictions aligned more strongly with the target next glimpse than with other same-scene glimpses, indicating sensitivity to spatial arrangement. (**C**) Recurrence supported integration across glimpses. Prediction loss was computed as the average cosine similarity to nontarget glimpses minus the cosine similarity to the target next glimpse, such that lower values indicate better prediction. Recurrent GPNs showed increasingly lower prediction loss over the glimpse sequence than their nonrecurrent counterparts, consistent with memory-based integration across glimpses. Error bars indicate 95% confidence intervals (CIs). Photo Credits: (A) DiamondBack Covers, Flickr, CC BY-SA 2.0 (https://creativecommons.org/licenses/by-sa/2.0/deed.en); “Frisbee fetch,” Paul (crimfants), Flickr, CC BY-SA 2.0 (https://creativecommons.org/licenses/by-sa/2.0/deed.en). Both images were cropped and annotated from the originals.

To quantify this learning of glimpse co-occurrence, due to training for glimpse predictions, we compared the alignment of the GPN predictions, given the first glimpse ($g_{0}$), with other glimpses from the same scene and with glimpses from the other scenes. Paired tests revealed a higher alignment for other glimpses from the same scene than glimpses from other scenes for all GPN variants [mean difference of cosines, $\bar{\Delta }>0.41$, Wilcoxon signed-rank tests: W=0, FDR-adjusted $P=5.9\times 10^{-8}$; N=25 batches of 206 glimpse sequences each; Fig. 2B]. Additionally, the cosine similarity of the GPNs’ prediction to the embedding of the input glimpse was much lower than *1* (1−μ>0.34, Wilcoxon signed-rank tests: W=0, FDR-adjusted $P=5.9\times 10^{-8}$), showing that the GPNs were not trivially implementing an identity mapping. Last, the prediction’s similarity to embeddings of glimpses from other scenes was close to zero (μ<0.002 for the GPNs, which was expected to be μ=0.37 assuming the “identity” mapping, constituting a baseline condition revealing effects expected given the cosine similarities among the input glimpse embeddings, by setting the GPN output = glimpse embedding input). These results suggest that GPNs learned a transformation that effectively orthogonalized glimpses from different scenes while maintaining similarity between glimpses within scenes, a sign that they accounted for the co-occurrence of glimpses.

To assess the dependence of learning of glimpse spatial arrangements on saccadic information, we compared the alignment of the GPN predictions, given the first glimpse ($g_{0}$), with the next, target, glimpse and with other glimpses from the same scene. If the cosine similarity of the GPN prediction with the target glimpse embedding is higher than its similarity with other glimpse embeddings from the same scene, then spatial arrangement is taken into account by the GPN. Checking across GPN variants, a 2 × 2 (recurrence × saccade presence) within-subject nonparametric contrast analysis revealed a main effect of saccades on the aforementioned difference between cosine similarities (W=0, Holm-adjusted $P=1.7\times 10^{-7}$; no interaction with recurrence, W=152, Holm-adjusted P=0.79; and no main effect of recurrence, W=97, Holm-adjusted P=0.16; Fig. 2B). After accounting for (i.e., subtracting), the cosine similarities expected from the identity baseline, we found better alignment with the next glimpse than other glimpses from the same scene for GPN-RS and GPN-S (averaged differences across variants; mean difference of cosines, $\bar{\Delta }=0.11$, W=0, FDR-adjusted $P=1.2\times 10^{-7}$), but not for GPN-R and GPN-B ($\bar{\Delta }=4.6\times 10^{-4}$, W=161, FDR-adjusted P=0.98). These results suggest that, with access to saccades, GPNs successfully learned a transformation over glimpse embeddings, accounting for the spatial arrangement of glimpses in scenes.

An important design aspect toward learning unified scene representations was the inclusion of recurrence, which was expected to enable GPNs to integrate information across glimpses in service of improving future glimpse predictions. Starting with the GPNs without recurrence, GPN-B and GPN-S, we observed that the prediction loss varied with glimpse number (Friedman tests, $\chi_{24}^{2}\geq 59$, FDR-adjusted $P=1.3\times 10^{-11}$) and was lowest for the first, central, glimpse (reflecting glimpse embedding statistics, captured by the identity condition). The addition of recurrence resulted in a prediction loss that was lower compared with the corresponding nonrecurrent GPNs, an effect that became increasingly evident with increasing glimpse number (Wilcoxon signed-rank comparisons over loss difference correlations with glimpse number; GPN-RS versus GPN-S: mean $\bar{\Delta }=0.79$, W=0, FDR-adjusted $P=1.1\times 10^{-7}$; GPN-R versus GPN-B: $\bar{\Delta }=0.39$, W=37, FDR-adjusted $P=3.3\times 10^{-4}$). These results suggest that, with access to recurrence, GPNs learned to use their memory states to drive information integration across glimpses in scenes, in the service of improving future glimpse embedding predictions.

To further characterize how GPN-RS integrates information across glimpses, we tested whether it learned a situated, content-location memory rather than relying only on natural spatial co-occurrence statistics. First, we trained cross-validated linear decoders to predict each glimpse’s absolute *x*, *y* location from model representations across layers and glimpse numbers (fig. S4A). In GPN-RS, location was decoded near ceiling from the first LSTM layer and remained available as glimpses accumulated, whereas this pattern was absent before training and in models lacking either recurrence or saccade inputs. This suggests that GPN-RS learned to path integrate the saccades and maintain a location-indexed recurrent state. We next tested whether this state could bind arbitrary content to locations by assigning glimpse embeddings from different scenes to fixation positions, thereby breaking natural co-occurrence and spatial-arrangement statistics, and simulating return saccades to previously visited locations (fig. S4B). GPN-RS retrieved the embedding previously encountered at the target location even for these artificial sequences, including when the embedding dimensions were independently shuffled. Thus, GPN-RS learned to construct a situated representation of what appeared where within the current sequence, rather than merely exploiting learned scene statistics. Because these arbitrary content-location bindings were formed online without weight updates, this ability can be viewed as a specific form of in-context learning, namely, in-context content-location binding (*18*).

Together, our analyses suggest that GPNs learned transformations that contextualized the glimpse embeddings by including: (i) across scenes, information about co-occurrence, and spatial arrangement (given saccades), of scene parts; and (ii) integrated information across glimpses seen in the current scene (given recurrence). Based on these observations, we consider that the sequences of glimpse embeddings were transformed into more holistic scene representations.

### GPN internal representations align with scene representations in visual cortex

Having established that GPNs encode co-occurrence, spatial arrangement, and information accumulated across glimpses, we next asked whether these learned representations explain cortical scene representations better than the input glimpse features, matched-objective control networks, and strong alternative models.

Scene representations were obtained from the Natural Scenes Dataset (NSD) (*19*). We considered the special-515 COCO scenes that all participants observed. GPNs were not trained on these scenes. To assess the alignment of the internal representations of the GPNs with representations in the visual cortex, we conducted representational similarity analyses (RSAs) (*20*). For each scene, we used one-sampled glimpse sequence. Across scenes, for a given glimpse number (*t*) in the sequence and a layer of a given GPN, including the input glimpse embedding as the first representational stage, we computed a representational dissimilarity matrix (RDM) across the 515 scenes and compared it with an RDM of a given region of interest (ROI) in the brain. Thus, each layer-by-glimpse-number pair yielded one GPN RDM for comparison with the corresponding ROI RDM. Alignment was assessed via nonnegative linear regression, as shown in Fig. 3A. For each GPN-ROI comparison, we summarized alignment as the maximum variance explained across layers and glimpse numbers. The same procedure was used for comparison networks, except that these networks had no glimpse-number dimension. The selected layers and glimpse numbers for the maximal alignment scores are reported in tables S1 to S7. We primarily focus on the alignment of GPN representations to the representations observed in the high-level ventral stream ROI, which subsumes high-level object and scene areas, such as the PPA (*21*) [“ventral” ROI from the NSD streams definition; hereafter termed ventral visual cortex (VVC)].

**Fig. 3.**
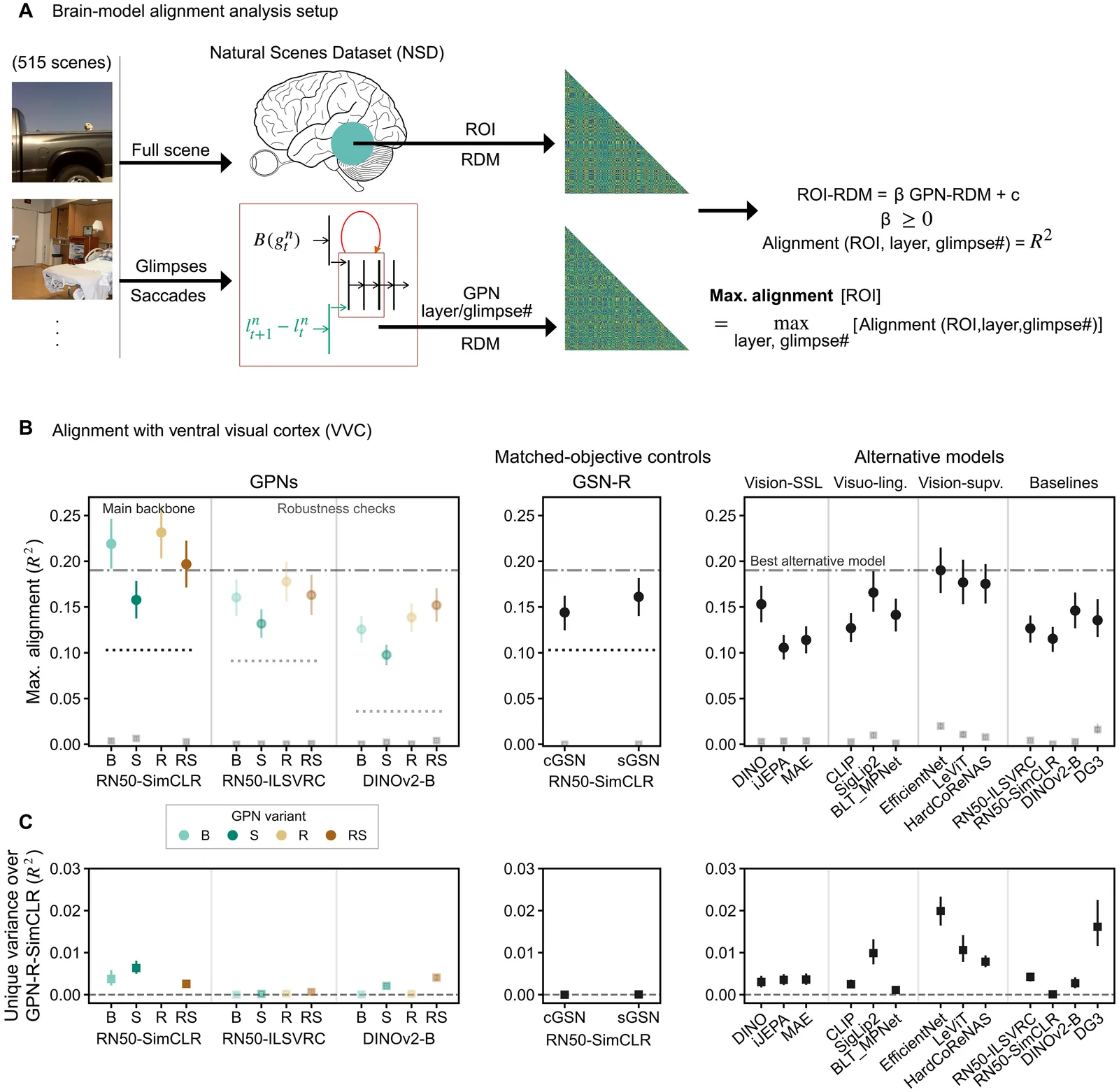
GPNs exhibit improved representational alignment with the scene representations in VVC, surpassing state-of-the-art models. (**A**) Representational dissimilarity matrices (RDMs) were computed from scene representations in a given ROI and the considered network. Alignment was quantified as the variance of ROI RDM explained by a network RDM via nonnegative linear regression. As the networks have multiple layers (and glimpse numbers in the case of GPNs), we report the maximum alignment across those layer/glimpse# comparisons per network. (**B**) Maximum alignment with the VVC, of the considered networks, is shown. Left: All GPNs, across variants and backbones, had higher alignment than their input glimpse embeddings (dotted black lines). Among the GPNs, GPN-R with the RN50-SimCLR backbone had the highest VVC alignment. Overall, recurrence helped alignment. Saccades hurt alignment over GPN-B. However, over GPN-R, they had mixed influence (as further described in fig. S6). Middle: GPN-R alignment was higher than glimpse stitching networks (GSNs) trained with the same architecture but different objectives: to predict the classes of objects in the scenes (cGSN) or semantic embeddings of scene captions (sGSN). Right: A large number of state-of-the-art models had lower VVC alignment than the RN50-SimCLR-based GPN-B and GPN-R (highest alignment across those models, for EfficientNet-B3, is indicated by the black line). (**C**) Moreover, other models explained little to no unique variance in the VVC RDMs over that explained by the RN50-SimCLR-based GPN-R [also indicated by square markers in (B)]. Error bars indicate 95% CIs. Photo Credits: (A) DiamondBack Covers, Flickr, CC BY-SA 2.0 (https://creativecommons.org/licenses/by-sa/2.0/deed.en); “P1090146,” Rick Kimpel, Flickr, CC BY-SA 2.0 (https://creativecommons.org/licenses/by-sa/2.0/deed.en). Both images were cropped from the originals.

We started by characterizing the influence of the various GPN components (saccades, recurrence, and backbones) on VVC alignment. For this, we computed the maximum alignment score for each GPN variant and three backbones (RN50-SimCLR, RN50-ILSVRC, and DINOv2-B) across all glimpse numbers in the sequences and layers (Fig. 3A; detailed results in tables S1 to S7). The degree of VVC alignment depended on an interaction of the inclusion of recurrence and saccades, and the backbone (2 × 2 × 3 repeated-measures analysis; three-way interaction: $\chi_{2}^{2}=14.3$, $P=8\times 10^{-4}$; Fig. 3B, top left). The addition of recurrence to GPN-B and GPN-S increased their VVC alignment (main effect; mean difference, $\bar{\Delta }=0.028$, W=0, Holm-adjusted P=0.02). The addition of saccades to GPN-B variants reduced their VVC alignment (averaged across backbones; $\bar{\Delta }=-0.039$, W=0, Holm-adjusted P=0.0078). However, the addition of saccades to GPN-R did not reliably increase or decrease the VVC alignment (decrease for RN50-SimCLR/ILSVRC: $\bar{\Delta }<-0.0147$, W=0, FDR-adjusted P=0.0078; increase for DINOv2-B: $\bar{\Delta }=0.013$, W=0, FDR-adjusted P=0.0078). We note that, for the best performing backbone, RN50-SimCLR, this negative influence of saccades was found throughout the brain, as indicated by a searchlight analysis that revealed GPN-R to be in better alignment than GPN-RS across the whole brain (fig. S5). A sweep across other well-known ResNet, convolutional, and transformer backbones supported these observations: Recurrence helped VVC alignment, but saccades hurt VVC-alignment over GPN-B, and the relationship between the addition of saccades to GPN-R and VVC alignment was not systematic (see fig. S6).

For each GPN variant and backbone, the best alignment score was higher than the maximum alignment of their input glimpse embeddings (denoted by black dotted lines in Fig. 3B, top left; across all GPNs, $\bar{\Delta }>0.04$, W=0, FDR-adjusted P=0.0078). These results suggest that the GPNs, taking the co-occurrence of scene parts into account and integrating information across them, did transform glimpse embeddings into scene-like representations aligned with representations of full natural scenes in the VVC.

Next, to assess whether the glimpse prediction objective, as opposed to the dataset (NSD images were also taken from COCO, which could lead to better alignment than the non-COCO trained backbone embeddings) and architecture (additional layers and recurrence, which could have increased expressivity), was essential for achieving the increased degree of representational alignment with VVC, we considered alternative objectives, keeping the architecture and dataset matched to the most VVC aligned, GPN-R with the RN50-SimCLR backbone. We term the resulting networks, glimpse stitching networks (GSNs), which incorporate two previously studied objectives (*7*). Classification-GSN (cGSN) was trained to categorize the objects in the scene, and semantic-GSN (sGSN) was trained to predict the semantic embeddings (MPNet based) of the corresponding scene captions. As seen in Fig. 3B (middle), both the GSN-R variants exhibited lower VVC alignment than GPN-R (cGSN: mean difference, $\bar{\Delta }=0.088$, W=0, FDR-adjusted P=0.0078; sGSN: $\bar{\Delta }=0.075$, W=0, FDR-adjusted P=0.0078). This result highlights the utility of the glimpse prediction objective, rather than the dataset or architecture changes, in acquiring scene representations aligned with the VVC.

To assess how the VVC alignment of the GPNs compared with related and state-of-the-art approaches, we benchmarked VVC alignment across a large number of alternative models. First, we considered self-supervised vision network families that were trained with objectives that fostered learning part-whole relationships by aligning representations across image parts (DINO, iJEPA, and MAE) (*22*–*24*). Second, we considered visuo-linguistic network families that were trained to align visual representations of scenes with representations of their corresponding captions (CLIP, SigLiP2, and BLT-MPNet) (*7*, *25*, *26*). Third, we considered network families with state-of-the-art alignment with occipitotemporal cortex (*27*) (EfficientNet, LeViT, and HardCoReNAS) (*28*–*30*). Fourth, we considered the three backbone variants used for GPN (RN50-ILSVRC, RN50-SimCLR, and DINOv2-B) and DeepGaze III (the generation of human-like fixations could involve human-like scene representation), as strong baselines. For each family of networks, we report the best observed alignment score across all networks and their corresponding layers in those families (Fig. 3B, top right). To account for the possibility that a central glimpse (approximating foveal vision, equivalent to glimpse 0 for the GPNs) might provide better alignment than the full scene (the typical input for these networks), given that the participants fixated centrally in the NSD experiment, we furthermore report the maximum alignment across the full scene versus central glimpse comparisons for all alternative models.

GPN-R with the RN50-SimCLR backbone achieved the highest VVC alignment among all tested networks (Fig. 3B, top). Among all alternative models tested, EfficientNet (B3 variant, trained for image classification on the ILSVRC dataset), presented with the central glimpse, was best aligned with VVC scene representations (alignment results for all the networks tested can be seen summarized in table S1 and detailed in tables S2 to S7). Among the self-supervised networks, a DINO-trained network (DINOv2-ViT-L14 variant) was the best aligned. Yet, GPN-B and GPN-R with the RN50-SimCLR backbone had significantly higher alignment than both of these highest-performing alternative models (mean differences across comparisons of GPN variants and backbones with the two best networks, $\bar{\Delta }>0.028$, W=36, FDR-adjusted P=0.016). In sum, the scene representations emerging due to the glimpse prediction objective, given the RN50-SimCLR backbone, surpassed the state-of-the-art models of scene representations in the VVC.

Although GPN-R, with the RN50-SimCLR backbone, explained the most variance in VVC among all the networks tested, it could have been the case that it explained different variance to the alternative models as the objectives, architectures, and training datasets vary substantially. To assess this possibility, we conducted variance-partitioning analyses, to assess whether the alternative models explain any unique VVC variance beyond that explained by GPN-R. As seen in Fig. 3C, the highest unique variance explained by any other network (over GPN-R), was observed for EfficientNet (μ=0.019, W=0, P=0.007). Yet, GPN-R accounted for 90% of variance explained by EfficientNet. The unique variance explained by the other networks was much lower, and we did not find evidence for the dataset/parameter-matched cGSN and sGSN explaining any unique variance ($\mu <4\times 10^{-4}$, W=0, FDR-adjusted P=0.33). In sum, GPN-R, with the RN50-SimCLR backbone, largely subsumed the variance explained in VVC by alternative, large-scale networks, which had access to the full scene images (during training and test) and, in some cases, access to rich semantic information through scene captions.

To test whether the high GPN alignment is specific to VVC, we extended our analysis to the other NSD streams ROIs: early, midventral, midlateral, lateral, midparietal, and parietal. First, for GPN-R, with the RN50-SimCLR backbone, the increase in alignment over input glimpse embeddings held across these ROIs ($\bar{\Delta }>0.012$, W=0, FDR-adjusted P=0.0078; fig. S7). Second, the GPN-R showed higher alignment than cGSN and sGSN across these ROIs (versus max GSN alignments, $\bar{\Delta }>0.0058$, W<2, FDR-adjusted P<0.015). Third, relative to the best-aligned alternative model for each ROI, the GPN-R was better aligned with the midparietal and parietal ROIs ($\bar{\Delta }>0.01$, W<3, FDR-adjusted P<0.05), similarly aligned with midventral, midlateral, and lateral ROIs ($\bar{\Delta }>-0.0075$, W>3, FDR-adjusted P>0.082) and worse aligned only with the early ROI ($\bar{\Delta }=-0.06$, W=0, FDR-adjusted P=0.023), understandably, as the alternative models could better capture low-level features, whereas GPNs begin with high-level glimpse embeddings. Last, in midparietal and parietal ROIs, where GPN-R surpassed alternatives, it explained ≥88% of the total variance explained by any other model. These results indicate that the glimpse-prediction objective yields scene representations aligning with large swaths of mid/high-level visual cortex, outperforming parameter/dataset-matched controls and matching or exceeding strong large-scale baselines, subsuming most of the variance they explain.

Having observed that GPN-R, with the RN50-SimCLR backbone, trained in a self-supervised fashion, achieved state-of-the-art alignment with ventral stream scene representation in NSD, we asked which layer, and hence computation, drives this effect. As seen in Fig. 4, the RDMs of the input glimpse embeddings (glimpse embed) accounted for ∼10% of the variance of VVC RDMs. For the first (central) glimpse, the LSTM output (lstm-out) nearly doubled this alignment (mean increase Δ=0.099, W=0, N=8, P=0.0078). With additional glimpses, due to recurrence, the alignment of lstm-out increased further (mean Spearman correlation between the alignment gain and glimpse number, ρ=0.97, W=0, P=0.0078). Notably, this increase in alignment was higher than expected by sequential averaging of glimpse embedding representations (glimpse-seqadd; reminiscent of building sentence embeddings from word embeddings; comparison of the maximum alignment of “lstm-out” versus “glimpse-seqadd”: $\bar{\Delta }=0.127$, W=0, P=0.0078). Critically, the best alignment was found at the LSTM output layer, which outperformed both the input and the prediction layers (e.g., at glimpse #5, the prediction layer alignment was lower than lstm-out; Δ=0.04, W=0, P=0.0078). Intermediate layers did not show comparable gains. We observed such maximum VVC alignment in the LSTM output layer across all the GPN variants and backbones (fig. S9; also for the midparietal/parietal ROIs, see fig. S8). In sum, GPNs transform input glimpse embeddings into a mid/high-level visual-cortex–aligned internal state, optimized for predicting the next-glimpse embedding.

**Fig. 4.**
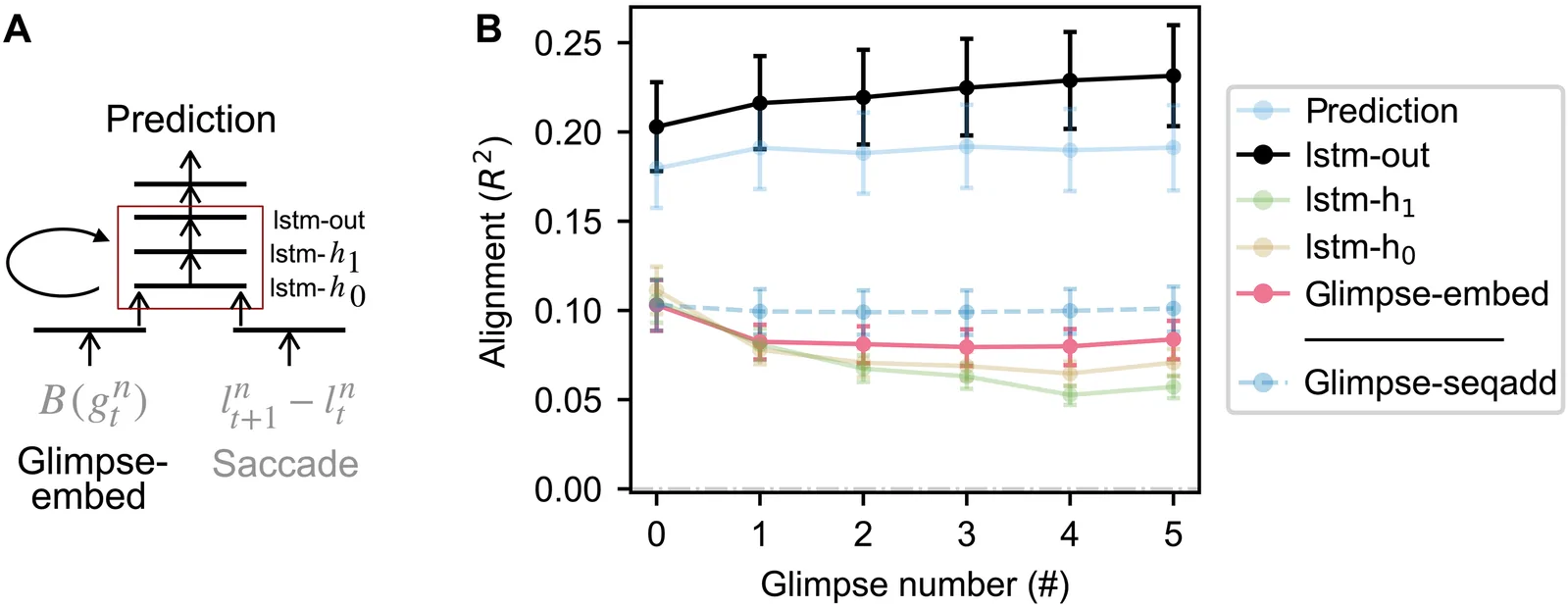
GPN-R transforms glimpse embeddings into a VVC-aligned recurrent state. (**A**) Layerwise analysis of the RN50-SimCLR-based GPN-R. We computed representational alignment with VVC for the input glimpse embedding, recurrent hidden states, LSTM output, prediction layer, and a sequential averaging baseline. Alignment was quantified as the variance of the VVC RDM explained by each model RDM via nonnegative linear regression. (**B**) The LSTM output showed the strongest VVC alignment. Input glimpse embeddings explained a portion of VVC representational structure, but the LSTM output substantially increased alignment from the first glimpse onward and improved further across the glimpse sequence. The glimpse-seqadd baseline was computed by sequentially averaging glimpse-embedding representations across the sequence, testing whether simple accumulation of viewed glimpse features was sufficient. LSTM-output alignment exceeded this baseline, indicating that recurrence did more than average the input glimpse embeddings. The prediction layer showed lower alignment than the LSTM output, suggesting that the most cortex-aligned representation emerges in the recurrent state optimized to support next-glimpse prediction. Error bars indicate 95% CIs.

## DISCUSSION

By taking stock of information available to the primate visual system, here we presented a self-supervised learning objective that predicts a high-level embedding of the next-glimpse in a sequence of human-like eye-movements. This approach, termed GPNs, resulted in networks that successfully internalize the co-occurrence of scene parts and their spatial arrangement and that integrate information across glimpses into a rich scene representation. These emergent representations outperformed their input-glimpse features in their alignment with scene representations across visual cortex, as well as GSNs trained to predict scene objects or caption embeddings. In mid/high-level visual cortex, these emergent representations equated or outperformed previously reported state-of-the-art ventral stream models, including large-scale foundation models.

The most intriguing finding of the current study was that the variance in VVC (as well as midparietal/parietal) scene representations explained by diverse models—supervised, self-supervised, vision only, and visuo-linguistic—was largely subsumed by that explained by the best-aligned GPN (GPN-R, with a ResNet50-SimCLR backbone). This suggests that the shared variance chiefly hinges on what this GPN-R captured: co-occurrence of scene parts and an integration across them. This convergence suggests a common structure for cortical scene representation alignment across current models (*31*). Notably, the results suggest that previously reported LLM-brain alignment (*6*, *7*) might be mediated primarily by co-occurrence structure, the same signals that underpin distributional semantics in language models that emerge through a similar objective: next-token prediction (*18*, *32*, *33*).

An unexpected result was that adding the saccade signal often reduced GPN alignment with NSD scene representations. This was clearest in the nonrecurrent models, where GPN-S aligned worse than GPN-B across nearly all backbones, but it also occurred for some recurrent models. This is unexpected because the same saccade information improved next-glimpse prediction, suggesting that better task performance does not necessarily imply better cortical alignment. Ventral stream responses are sensitive to object co-occurrence (*34*, *35*) and spatial arrangement (*36*), and the parietal ROI overlaps with PPC/IPS regions sensitive to relative position and configural information (*37*, *38*). Still, alignment did not consistently improve when GPNs were given explicit spatial information through saccades. One possibility is that, because GPN-B does not receive explicit saccade information, it can only solve the next-glimpse prediction task by learning more location-agnostic scene associations: Given one glimpse, what other visual content is likely to occur in the same scene? This kind of location-agnostic association may better match centrally fixated NSD responses than the more specific object-location predictions enabled by GPN-S, even if the latter are useful for next-glimpse prediction. The recurrent models complicate this picture: GPN-R integrates content across glimpses without explicit location information, whereas GPN-RS can use recurrence and saccades to maintain what appeared where across the sequence. This may allow GPN-RS to move from single location-conditioned prediction toward an activity-based memory of multiple glimpse-location bindings, but whether this helps or hurts cortical alignment was backbone dependent. Thus, the reduction in alignment may reflect limited spatial-arrangement information in NSD responses, a mismatch between GPN and cortical spatial codes, or a difference between location-agnostic scene association and location-specific glimpse prediction. Disambiguating these accounts is an important next step toward improving models of cortical scene representation, which, despite achieving state-of-the-art alignment, still explain less than half of the variance shared across participants.

In sum, by highlighting the utility of predicting upcoming features during eye movements, our results point toward the next generation of self-supervised models of human cortical scene representation: networks based on the general principle of discovering the structure of the world through temporal, sensorimotor predictions (*13*, *39*–*41*). The success of GPNs originates from taking stock of the signals available to the visual system, rather than following the “computational opportunism” of testing large-scale models engineered toward AI performance, and thereby underlines the importance of cognitive computational neuroscience to emancipate itself, at least to a degree, from AI engineering (*42*).

## MATERIALS AND METHODS

### Network architecture

#### Glimpse prediction networks

Given an ordered sequence of fixations and the associated glimpse $l_{i}$, $g_{i}$, and a backbone *B*, GPNs predict the backbone’s embedding of the next glimpse, i.e.

$$B{\left(g_{t+1}\right)}^{\text{prediction}}=\text{GPN}\left[B\left(g_{t}\right),S\times \left(l_{t+1}-l_{t}\right),R\times \left(h_{t-1}\right)\right]$$

where *S* controls if the saccade vector (interglimpse displacement) is available and *R* controls if the previous hidden state is available. If *R* is true, GPN contains an LSTM with three layers with hidden size of 1024 (implemented with the PyTorch LSTM class) (*43*). If false, it instead contains the forward pass of the same LSTM with the recurrent components set to zero. The glimpse embedding and saccade vector were transformed to 512D each, concatenated, and passed on to the LSTM. The LSTM output was projected to a 341D hidden layer, from which a projection served as the prediction. Layer-norm was used on all layers except the LSTM layers (but it was used on the LSTM output). Dropout was used in all the glimpse representations and in the LSTM. ReLU was used on all non-LSTM layers except the prediction layer.

GSNs used the same architecture, but different prediction output sizes, corresponding to their targets, caption embedding for sGSN and multihot object classification for cGSN. The GSNs predicted their scene-level targets given each glimpse embedding input in the sequence, similar to the GPNs predicting the next glimpse embedding per glimpse embedding input. Additionally, for cGSN, which predicts multihot labels of objects in the COCO scenes, no layer-norm was used on the prediction layer. The “stitching into a target representation, using recurrent networks” process was inspired by previous work (*44*).

Specifically, here, we considered cGSN-R and sGSN-R with SimCLR backbones, which are comparable on architecture and training data to GPN-R, with the RN50-SimCLR backbone, to assess the influence of objective on the alignment of the internal representations of these networks to the human visual cortex.

#### GPN architecture search

The aforementioned architectural configuration was decided by running a hyperparameter sweep over the #layers (*1*, *3*, *5*) and hidden size (512, 1024, 2048) of the LSTM, and the input ($d_{\text{in}}$) and LSTM dropout ($d_{\text{lstm}}$) parameters (0, 0.1, 0.25, 0.5) to optimize the prediction loss for GPN-RS-ILSVRC on the “train”/“val” sets. For all the other GPNs (and for the GSNs), the dropout parameters alone were optimized for lowering prediction loss on the train/val set. The dropout parameters chosen for each GPN/GSN are listed in table S2. Final training was performed on the larger “train-515” set, and evaluation was carried out on the test-515 set.

To verify the validity of the trained GSNs, we assessed the alignment of their internal representations to scene representations in VVC. First, as seen in Fig. 3, we note that the GSNs replicated a previously reported finding: The caption embedding prediction objective (sGSN) yielded better alignment with VVC than the object class prediction objective (cGSN; mean difference, $\bar{\Delta }=0.017$, W=0, P=0.0078) (*7*). Additionally, the degree of VVC alignment of sGSN was comparable (slightly higher, $\bar{\Delta }=0.02$, W=0, p=0.0078) to the previously reported alignment based on a different recurrent architecture trained only on full COCO scenes (BLT-MPNet).

### Network objectives

#### Contrastive prediction loss

A contrastive prediction loss was used to train the GPNs, as shown in Fig. 1A. The positive and negative pairs were sampled from a batch of glimpse sequences from randomly selected scenes (ordered to form an epoch). Cosine similarity to the target glimpse embedding was maximized, whereas the cosine similarity to the embeddings of other glimpses from the same sequence and glimpses from other sequences was minimized, with equally shared weighting of the average similarities. To avoid minimizing similarity to instances of glimpse refixation, we filtered out negative pairs with cosine similarities higher than 0.999.

For sGSN, a cosine similarity loss was used to maximize the alignment to the target caption embedding and minimize the alignment to other scenes’ caption embeddings. For cGSN, binary-cross-entropy loss with the multihot target vectors was minimized.

#### Training details

The learning rate was reduced on the plateau of the training loss, until it reached 10^−8^, when training was stopped. However, before this, if validation loss increased more than 1% from the minimum validation loss recorded, then training was stopped. The checkpoint with the lowest validation loss was saved.

### Image datasets

#### COCO scenes and glimpses

Scene images were taken from the 2017 train split of the COCO dataset. Central crops (256 px) were considered after scaling the smallest side of each image to 256 px. DeepGaze III was used to sample 10 fixation sequences for each scene (both the order of fixations and the prior distribution of fixations across scenes positively influenced the VVC-alignment results for GPN-R, with the RN50-SimCLR backbone, shown in Fig. 3; see fig. S1). Each sequence contained seven fixations, all starting at the center of the images. Glimpse crops (91 px) were extracted centered on these fixations, corresponding to a 3° window (approximately the full-width at half-maximum of retinal cone density) (*45*), assuming the image extended 8.4° of visual field (corresponding to the display parameters from NSD). Semantic embeddings of the captions of the COCO scenes were obtained through MPNet (768D) (*7*). Multihot labels signaling the presence of 91 object classes in the scenes were also obtained from the COCO dataset.

DeepGaze III and the visual backbones were pretrained and frozen throughout; only the GPN or GSN parameters were optimized in the experiments reported here. The data were split into three sets: “train” with ∼43,000, “val” with ∼2000, “test” with the ∼73,000 COCO scenes that are part of NSD (*7*). We also created two more splits, train-515 with ∼115,000 scenes (pooling from train and test) and test-515 with the special-515 scenes that each participant saw thrice in NSD. The original, smaller, train/val splits were used for running fast hyperparameter sweeps. The train-515 split was created to train GPNs/GSNs with the maximum amount of data available that does not overlap with the test set of interest, the special-515 scenes from NSD.

#### Backbone feature encoders

Three encoders were used to extract high-level embeddings of these glimpses: Resnet-50 trained on ILSVRC dataset to classify images (v1) (*46*) and one trained with SimCLR (*47*), and a ViT trained with DINOv2 on LVD-142M (v2, B/14) (*48*). The AvgPool layer responses of the ResNets (2048D) and the output embedding of DINO (768D) were used as glimpse embeddings.

Prediction was conducted in high-level embedding space, as opposed to directly on glimpse pixels, as pixel-based prediction would require a much larger generative network and has been shown to rely on features uninformative for high-level perception of objects (*49*, *50*). Pretrained backbones were used, as training the backbone with our procedure did not yield meaningful behavioral and neural alignment results, possibly owing to poor feature learning due to low diversity of the COCO images (∼115,000 images), as compared with ILSVRC (∼1 million images) or LVD-142M images that the backbones were trained on.

### Behavioral analysis

To assess whether the GPNs were sensitive to co-occurrence and spatial arrangement of glimpses in natural scenes and whether they could integrate information across glimpses to improve future predictions, we compared the GPN predictions with the relevant target glimpse embeddings. For example, a sensitivity to co-occurrence was indicated by the prediction being better aligned (higher cosine similarity) with embeddings of glimpses from the same scene than from other scenes. These comparisons were conducted within batches of glimpse sequences over the test-515 scenes. In total, we used 5150 glimpse sequences (10 per scene), which were distributed randomly into 25 batches of 206 glimpse sequences each.

### Brain alignment analysis

#### Natural Scenes Dataset

Neural scene representations were acquired from the NSD. Each of the eight participants saw up to 10,000 scene images, performing a long-term continuous recognition task. For our analyses comparing GPNs and other models to brain representations, we used the set of 515 scene images that all eight participants saw three times. Each of the eight participants’ betas (from condition-tied GLMs conducted on the BOLD responses) were transformed to the “fsaverage” space, and betas were acquired from seven ROIs from the streams definition, provided in NSD: early, midlateral, midventral, midparietal, ventral, lateral, and parietal. Their definitions, from the NSD manual, can be found in the Supplementary Materials. Betas were averaged across those repeats to get more robust estimates of neural response. Note that the NSD data were collected under the original study’s institutional ethics approval and informed-consent procedures; no new human-subject data were collected for this study.

#### Alignment based on linear regression

To assess the representational alignment between the representations from a brain ROI and a network layer, RSA was used, which quantifies the alignment between representational geometries, i.e., the alignment of principal dimensions of representational variation. RDMs were computed from the ROI betas and from the network layer, using pairwise Pearson correlation distances, for the special-515 scenes. The alignment was computed using nonnegative linear regression (scikit-learn, positive=True) (*51*), as shown in Fig. 3A. Specifically, the lower triangle of each RDM was flattened, standardized (μ = 0, σ = 1), and the linear regression parameters were fitted to minimize the squared error with respect to the flattened lower triangle of the ROI RDM. This choice was made to make the alignment readily amenable to the variance partitioning analysis explained next. The pattern of results looked similar when using Pearson correlation between the brain and model RDMs (fig. S10; which is typically used in RSA) (*20*).

#### Variance partitioning analysis

Unique variance of an ROI RDM (A) explained by an alternative network RDM (B), compared with variance explained by a GPN RDM (C), is computed as the variance of A explained jointly by B and C minus the variance of A explained by C alone. The variance explained is computed using nonnegative linear regression.

It is important to note that this procedure likely overestimates the unique variance explained by models beyond GPN-R (with the RN50-SimCLR backbone): In multiple regression with correlated predictors, “suppression” effects can inflate the total variance explained by the joint model (*52*). However, in our case, the estimated unique contributions of the alternative models are already very small, so any downward correction would only strengthen our conclusion that they add little beyond the GPN-R.

#### Subject shared variance

Per ROI, we computed an estimate of the shared variance across subjects as a benchmark of how much variance a single model could capture across subjects in NSD: Iteratively, we computed the variance explained (via nonnegative linear regression) in the left-out-subject’s RDM by the average RDMs of *N −* 1 subjects and reported the average explained variance. This corresponds to the lower bound of the noise ceiling in typical RSA frameworks.

#### Alternative models

Brain alignment of the GPNs was compared with a large number of related and state-of-the-art approaches. For each family of networks, we considered openly available small, medium-sized, and the largest variants. Per ROI, alignment was computed with the RDMs over the special-515 scenes (and also their central 91-px crops) per network variant and layer. The best alignment score across variants and their layers was associated with the network family. All the network families and variants tested and their layers with the best alignment scores to ventral ROI from NSD are summarized in table S1 and detailed in tables S2 to S7.

### Statistical analyses

We performed all comparisons on bounded similarity metrics, cosine similarity ([−1,1]) and variance explained ($R^{2}\in \left[0,1\right]$), using nonparametric tests. Unless stated otherwise, tests were two-sided with α=0.05.

#### Paired two-level or one-sample tests

We used the Wilcoxon signed-rank test (SciPy) (*53*). We report the Wilcoxon *W* statistic (sum of the ranks of the differences above or below zero, whichever is smaller) and the *P* value. Zero differences were excluded (SciPy’s default), and exact *P* values were used when available; otherwise the large-sample approximation with continuity correction was used.

#### >2 levels (within-subject)

We used the Friedman test (SciPy), reporting $\chi_{\text{df}}^{2}$ and the *P* value.

#### Hierarchical 2 × 2 × N design (recurrence × saccade × backbone)

For each subject we formed interaction summaries as the average contrast

$$\frac{\left(\mathrm{RS}-R\right)-\left(S-B\right)}{2}$$

and tested whether these differed across backbones using the Friedman test. Because the three-way interaction was significant in our data, we did not proceed to simple-effects follow-ups. Main-effect summaries were formed as averages over the other factor, e.g., for recurrence

$$\frac{\left(R-B\right)+\left(\mathrm{RS}-S\right)}{2}$$

and assessed with Wilcoxon signed-rank tests; *P* values across main-effect summaries were Holm-adjusted. When *N* = 1, we tested the interaction and both main-effect summaries with Wilcoxon signed-rank tests and applied Holm correction across the three tests.

#### Multiple comparisons correction

When multiple post hoc/independent Wilcoxon/Friedman tests were conducted simultaneously (e.g., several layers in one analysis; data a priori independent), we controlled the false discovery rate with Benjamini-Hochberg (statsmodels) (*54*). Instead, when correcting a small, prespecified set of planned contrasts (e.g., main-effect/interaction summaries; data a priori shared and restructured), we controlled the familywise error rate with Holm adjustment (statsmodels).

## Supplementary Materials

### This PDF file includes:

Figs. S1 to S11 Tables S1 to S7 References
