# Supplementary material for: Predicting upcoming visual features during eye movements yields scene representations aligned with human visual cortex
Source: Sci Adv. 2026 Oct 21;12(43):eaee1955. doi: 10.1126/sciadv.aee1955 (PMC13685946; doi:10.1126/sciadv.aee1955)
Supplement: Supplementary file 1 [file sciadv.aee1955_sm.pdf]

Science Advances
AAAS

Supplementary Materials for

Predicting upcoming visual features during eye movements yields scene representations aligned with human visual cortex

Sushrut Thorat *et al.*

Corresponding author: Sushrut Thorat, sthorat@uos.de; Tim C. Kietzmann, tkietzma@uos.de

Sci. Adv. **12**, eaee1955 (2026)
DOI: 10.1126/sciadv.aee1955

This PDF file includes:

Figs. S1 to S11
Tables S1 to S7
References

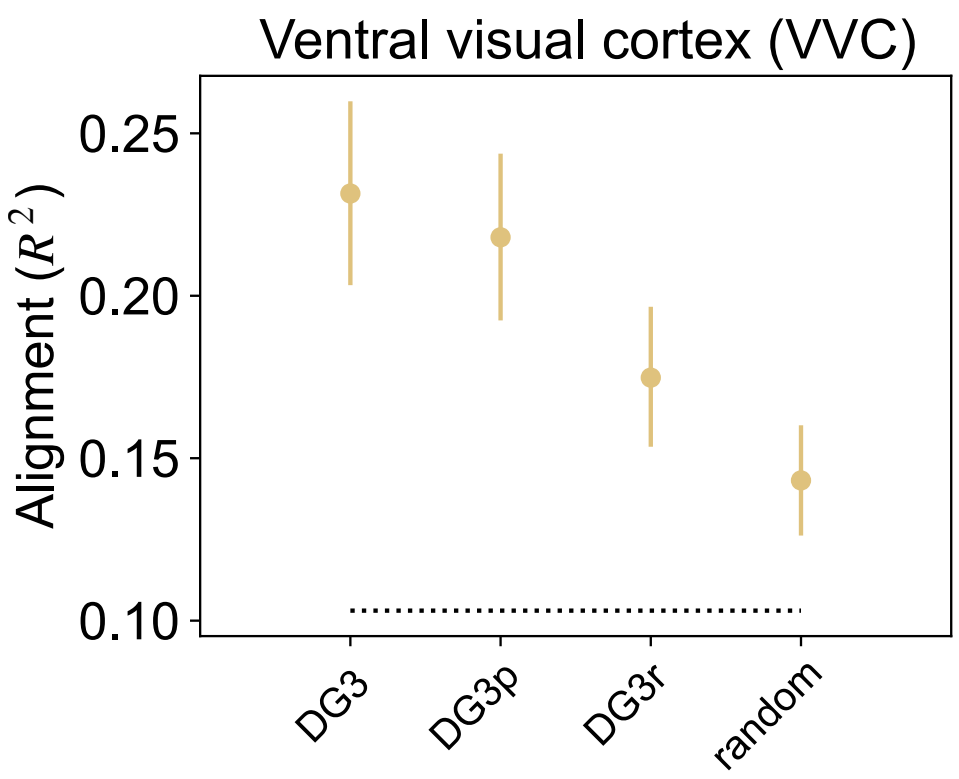

Figure S1: **VVC-alignment of variants of GPN-R, with the RN50-SimCLR backbone, trained with differing glimpse sequences.** For GPN-R, glimpses were sampled using DeepGazeIII (DG3). To assess the importance of DG3 fixations in generating VVC-aligned scene representations, seen in Fig. 3, we considered three more conditions. (i) DG3p: within each fixation sequence, the fixation order was permuted (barring glimpse 0), (ii) DG3r: the fixation locations per sequence were randomly sampled from all possible fixation locations across the scenes (barring glimpse 0), capturing the prior distribution of DG3 fixations (center-biased), and (iii) random: the fixation locations were sampled uniformly-randomly from the scenes (barring glimpse 0). Six pairwise comparisons revealed that (i) the prior center-biased fixation distribution of DG3 was important to acquire VVC-aligned scene representations (DG3r - random; mean difference, $\bar{\Delta} = 0.031$, W = 0, FDR-adjusted p = 0.0078); (ii) scene-conditioned fixation distributions increased the alignment (DG3p - DG3r; $\bar{\Delta} = 0.043$, W = 0, FDR-adjusted p = 0.0078); and (iii) the sequence of scene-conditioned fixations further increased alignment (DG3 - DG3p; $\bar{\Delta} = 0.013$, W = 0, FDR-adjusted p = 0.0078). Notably, the variant with random glimpses still improved alignment with VVC over the max-alignment of input glimpse embeddings (for the central glimpse; dotted line; $\bar{\Delta} = 0.04$, W = 0, p = 0.0078). These results suggest the glimpse prediction objective improved VVC-alignment, even when random glimpses were provided. However, the use of DeepGazeIII fixation sequences was critical to the high VVC-alignment of emergent scene representations in GPN-R.

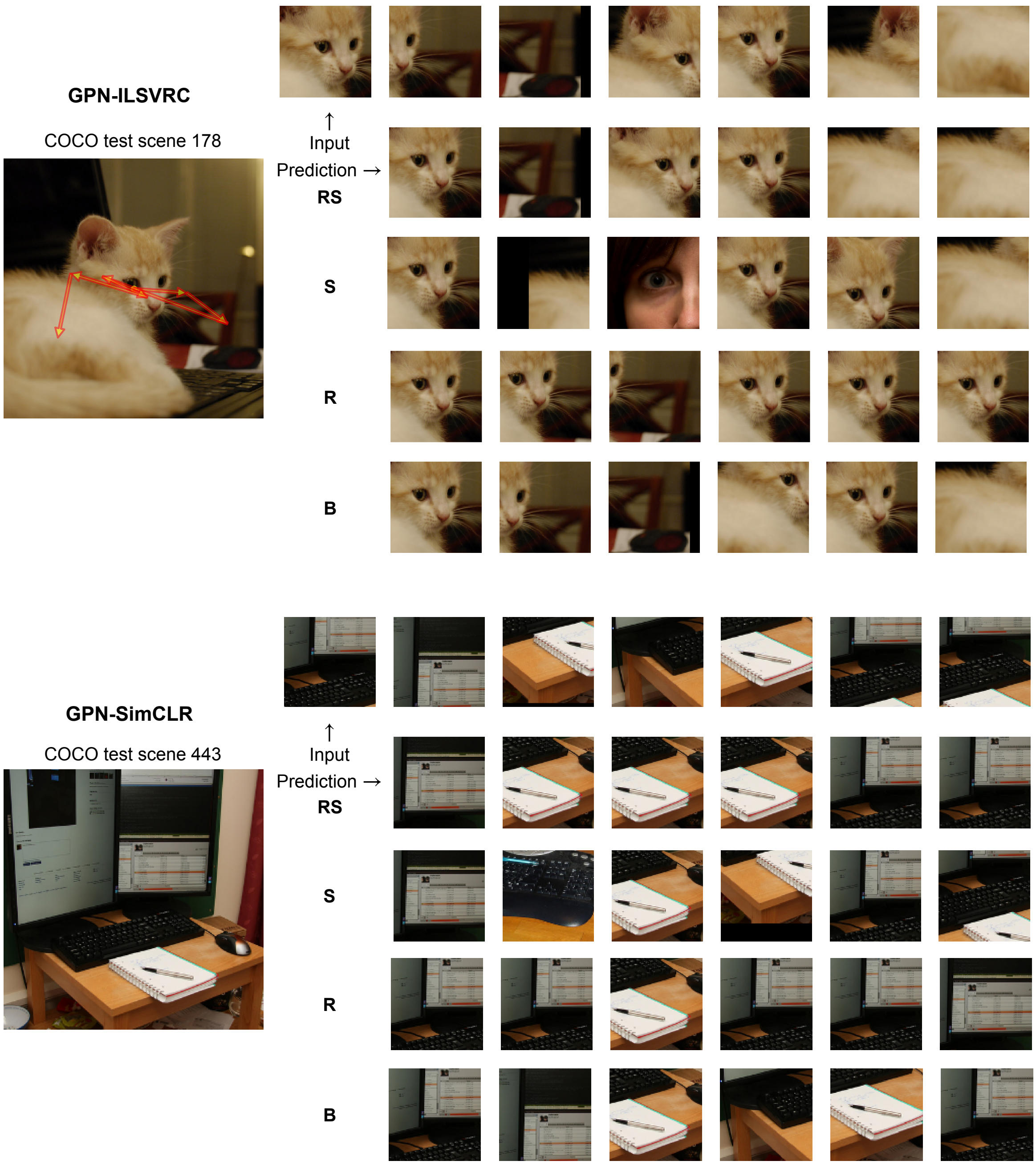

Figure S2: **Qualitative assessment of the behavior of GPNs across variants and backbones** (see Fig. 2A for reference). Photo Credits: “Samouraï, futur geek”, Camille Gévaudan, Flickr, CC BY-SA 2.0; “Standing desk + Das Keyboard”, Adam Greig, Flickr, CC BY-SA 2.0. Both images were cropped from the originals.

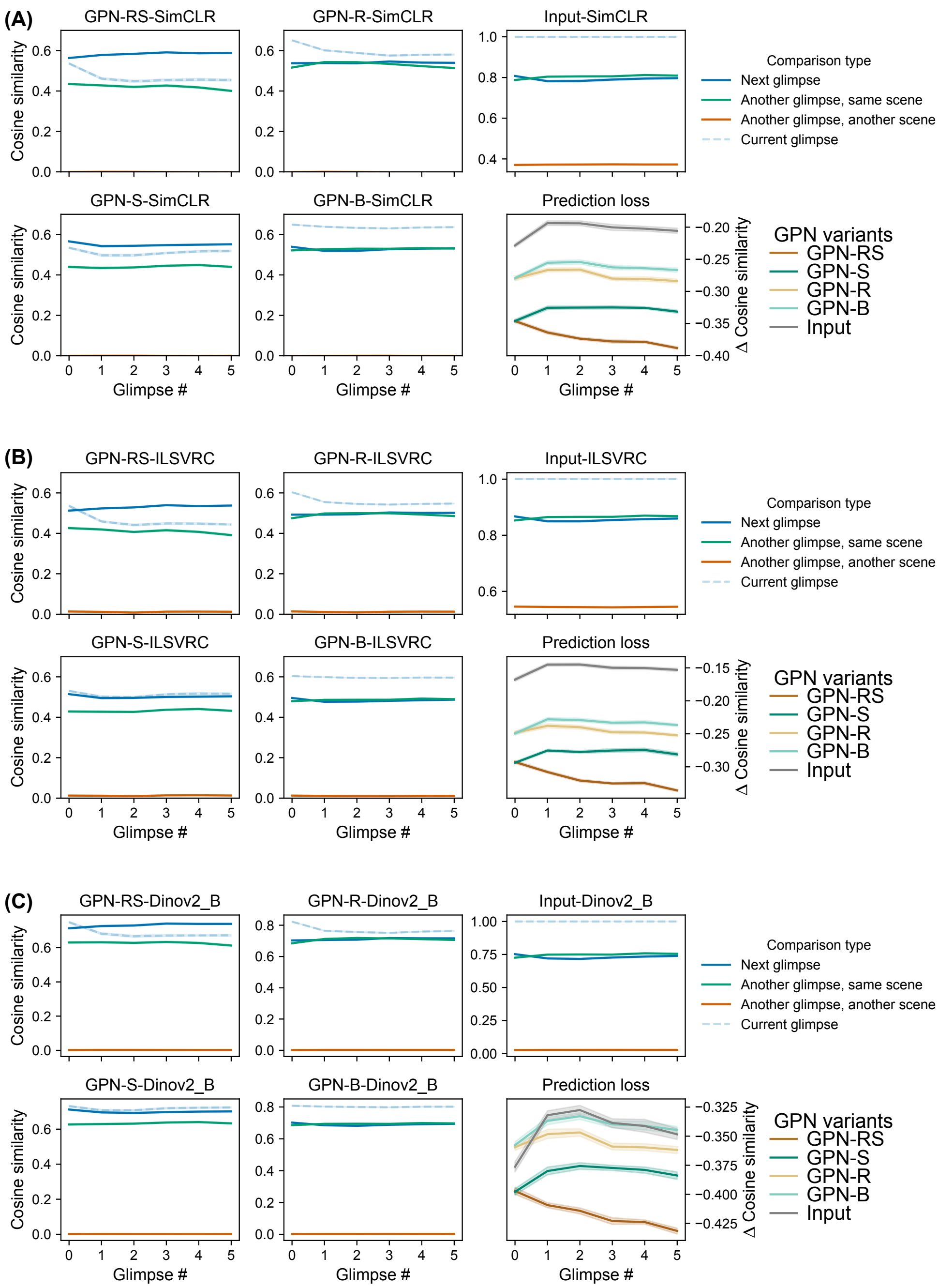

Figure S3: **Quantitative assessment of the behavior of GPNs across variants** (see Fig. 2B for reference) for, **(A)** the SimCLR backbone, **(B)** the ILSVRC backbone, and **(C)** the DINOv2-B backbone.

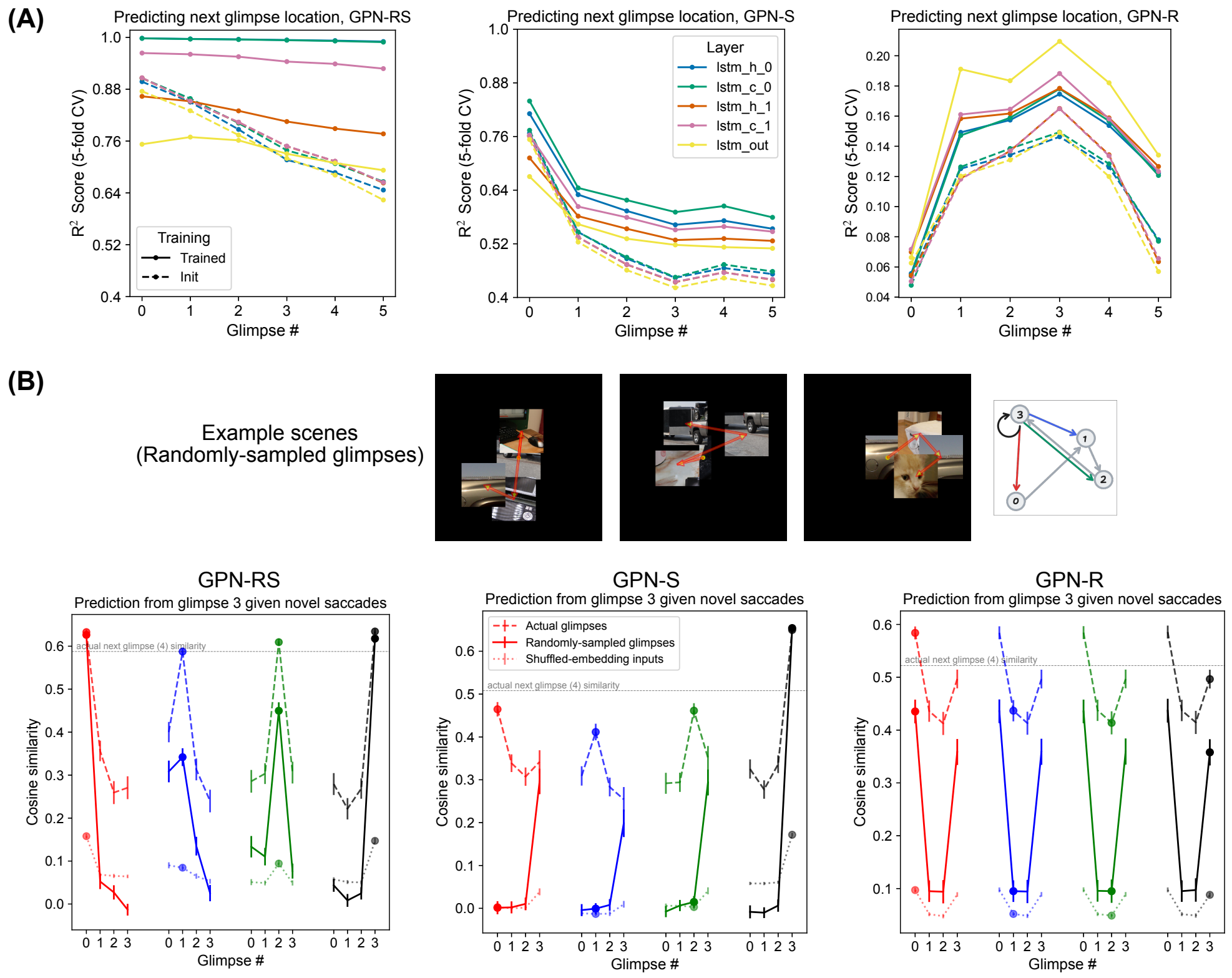

Figure S4: **Behavioral assessment of glimpse integration (SimCLR backbone).** As shown in Fig. 2B-C, GPN-RS leveraged spatial arrangement and integrated across glimpses to improve future-glimpse prediction. A plausible mechanism is the path integration of saccades that anchors glimpse embeddings to absolute locations, i.e. tracking what is where. (A) We fit 5-fold cross-validated linear regressions to predict absolute position (x,y; R^2 averaged across axes) from model representations across layers and glimpse numbers in the sequences. In GPN-RS, variance explained in the first LSTM layer stayed near 1 as glimpses accrued, consistent with path integration. This effect was absent in the initialized GPN-RS ('Init') and in GPNs-R/S after training, indicating that recurrence and saccades are required to learn path integration. (B) Path integration implies that refixations should cue the correct content at that location. We tested three conditions over 1,000 fixation sequences (first four fixations) from the test-515 set (example scenes are depicted), simulating refixations from glimpse #3 toward each of the four locations (setup inset shown on the right): (i) Actual glimpses (natural co-occurrence/arrangement), (ii) Randomly-sampled glimpses (glimpse embeddings across scenes randomly assigned to fixations to violate co-occurrence and arrangement), and (iii) Shuffled-embedding inputs (embedding dimensions independently shuffled per glimpse to destroy natural glimpse features). GPN-RS retrieved the correct target embedding under all three conditions; GPN-S succeeded only for 'Actual glimpses', as expected without recurrent integration. GPN-R could not succeed for any condition, as expected without saccadic inputs. Together, these results indicate that GPN-RS learned a general embedding-location binding mechanism (*55*). Error bars in (B) show 95% CIs. Photo Credits: "Samouraï, futur geek", Camille Gévaudan, Flickr, CC BY-SA 2.0; DiamondBack Covers, Flickr, CC BY-SA 2.0; "Frisbee fetch", Paul (crimfants), Flickr, CC BY-SA 2.0; "4 Students 4 Phones", Alan Levine, Flickr, CC BY-SA 2.0; "P1090146", Rick Kimpel, Flickr, CC BY-SA 2.0; "Standing desk + Das Keyboard", Adam Greig, Flickr, CC BY-SA 2.0. All images were cropped and composed from the originals.

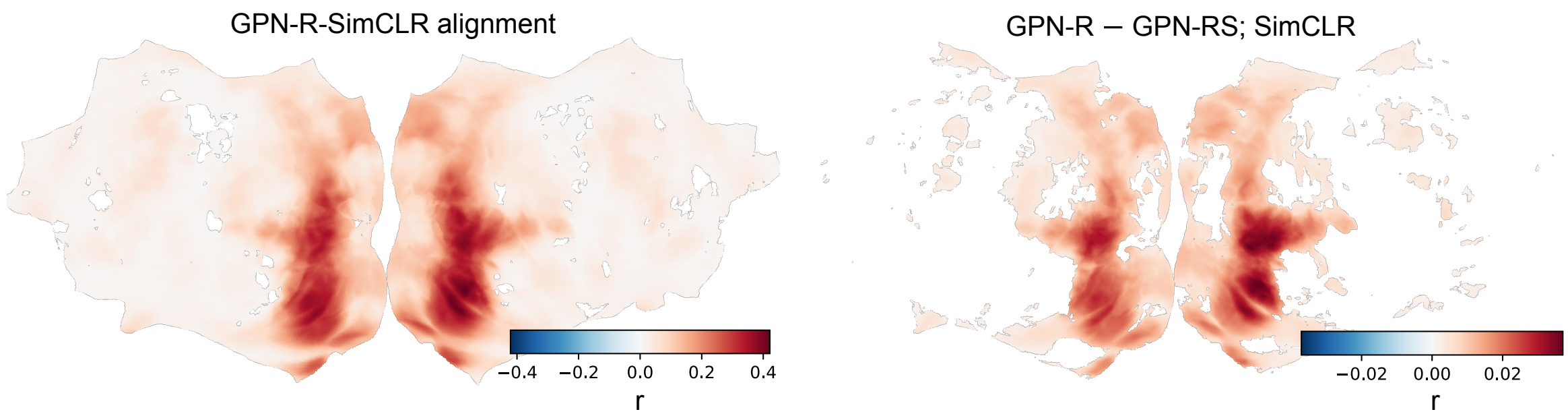

Figure S5: **Searchlight analysis to compare the alignment of GPN-R vs GPN-RS (RN50-SimCLR backbone) across the brain.** Searchlight spheres with a radius of 6 voxels, i.e. covering 900 voxels, were used. Pearson correlation was used to quantify alignment. No spheres revealed a higher alignment with GPN-RS than with GPN-R. Correlations for spheres that were significant ($p < 0.05$; FDR-adjusted across the brain) are shown.

Table S1: **Overview of network families tested for VVC alignment.** The detailed model-level alignment results are split across Tables S2–S7. Each detailed table reports the best-aligned input/layer and the average VVC alignment.

Model family	Models included	Purpose	Details
GPNs and matched controls	GPN variants across RN50-ILSVRC, RN50-SimCLR, and DINOv2-B; cGSN-R-SimCLR; sGSN-R-SimCLR	Proposed models and matched objective controls	Table S2
DINO-family SSL vision models	DINOv2, DINO-WebSSL, and DINOv3 variants	Strong vision-only self-supervised alternatives	Table S3
iJEPA and MAE SSL vision models	iJEPA and MAE variants, including WebSSL MAE models	Additional predictive and reconstruction-based SSL alternatives	Table S4
Supervised and architecture-optimized vision models	EfficientNet, LeViT, and HardCoreNAS variants	State-of-the-art visual model families	Table S5
Visual baselines	RN50-ILSVRC, RN50-SimCLR, and DeepGazeIII	Backbone and fixation-related baselines	Table S6
Vision-language and semantic baselines	CLIP, SigLIP2, and BLT-MPNet	Language- or semantic-supervision baselines	Table S7

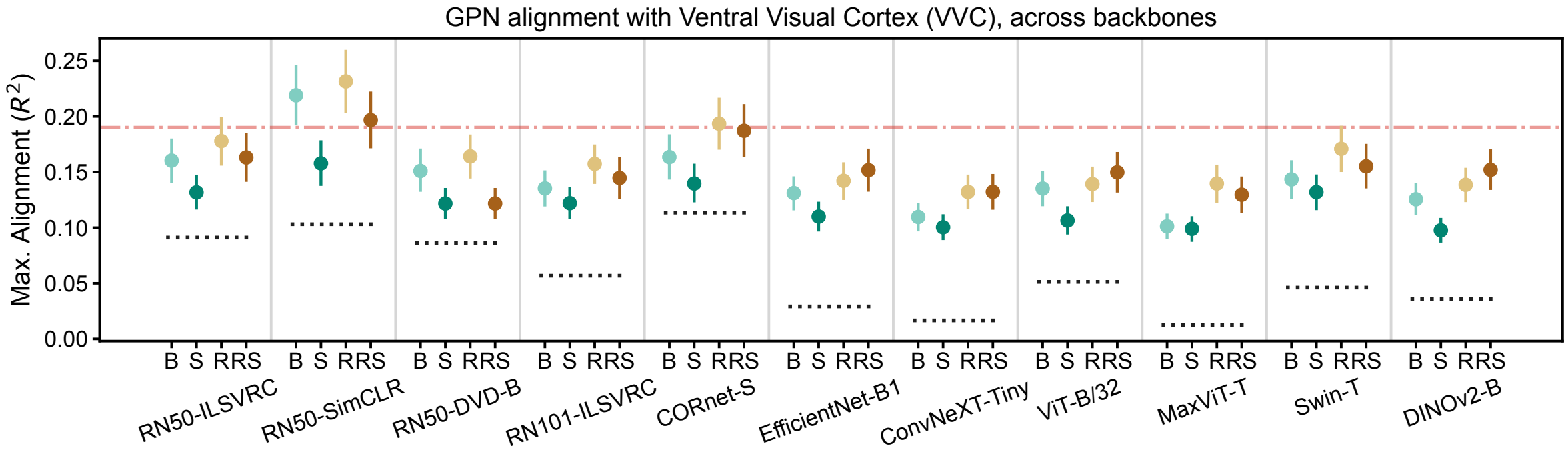

Figure S6: **VVC-alignment of GPN variants trained over different backbones.** In Fig. 3B, we saw that with the ResNet backbones, GPN-RS had a lower VVC-alignment than GPN-R, whereas with the DINO backbone, GPN-RS had a higher VVC-alignment than GPN-R. To shed further light on this RS vs R effect as a function of backbone, we trained GPNs with eight more backbones. This included two more ResNet backbones, one trained on ILSVRC (RN101-ILSVRC) and the other trained on an infant-like curriculum using Ecoset images (RN50-DVD-B) which yields a shape-biased model (*56*). It further included a recurrent convolutional neural network (CNN; CORnet-S), which is one of the best models of ventral stream object representations (*57*), two more CNNs (EfficientNet-B1 and ConvNeXT-Tiny (*58*)), and three more vision transformers (ViT-B/32, MaxViT-T, and Swin-T (*59–61*)). The degree of VVC-alignment depended on an interaction of the inclusion of recurrence and saccades, and the backbone ($2 \times 2 \times 11$ repeated-measures analysis; 3-way interaction: $\chi^2_{10} = 76.1$, $p = 3 \times 10^{-12}$). The inclusion of recurrence on top of GPN-B or GPN-S increased VVC-alignment across all backbones ($\bar{\Delta} > 0.01$, $W = 0$, FDR-adjusted $p = 0.0086$), except for GPN-S with the DVD-B backbone where there was no difference ($\bar{\Delta} = 0.0$, $W = 0$, FDR-adjusted $p = 1.0$). In contrast, for all backbones, except MaxViT-T, the inclusion of saccades on top of GPN-B reduced VVC-alignment ($\bar{\Delta} < -0.009$, $W = 0$, FDR-adjusted $p = 0.0086$; for MaxViT-T, $\bar{\Delta} = -0.002$, $W = 7$, FDR-adjusted $p = 0.15$). However, the inclusion of saccades on top of GPN-R had a mixed effect: all the ResNet, CorNet-S, MaxViT-T, and Swin-T backbones showed reduced VVC-alignment ($\bar{\Delta} < -0.006$, $W = 0$, FDR-adjusted $p = 0.0086$), whereas the EfficientNet-B1, ViT-B/32, and DINOv2-B/14 backbones showed increased VVC-alignment ($\bar{\Delta} > 0.009$, $W = 0$, FDR-adjusted $p = 0.0086$), and the ConvNeXT-Tiny backbone showed no difference ($\bar{\Delta} = 6 \times 10^{-5}$, $W = 16$, FDR-adjusted $p = 0.84$). These results suggest that the reduction in VVC-alignment with saccade inclusion over GPN-R was not specific to the ResNet backbones, and we could not identify any clear pattern.

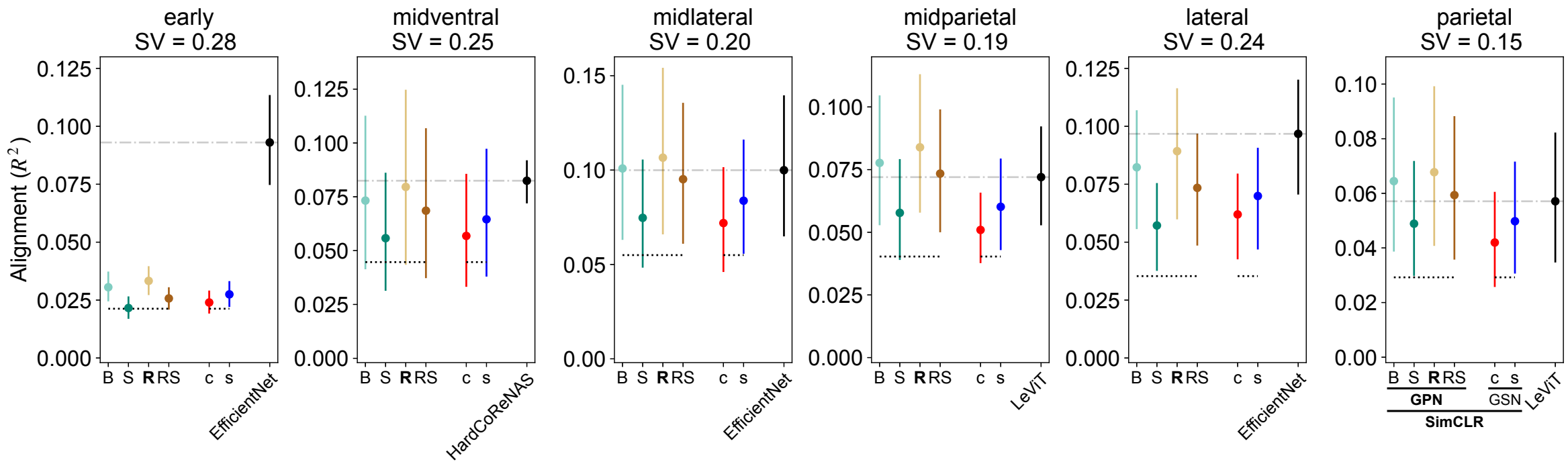

Figure S7: **Alignment of GPNs with SimCLR backbone and comparison networks across the streams ROIs** (see Fig. 3 for reference). Per ROI, the alignment of the GSNs and the best-aligned alternative network for that ROI are shown.

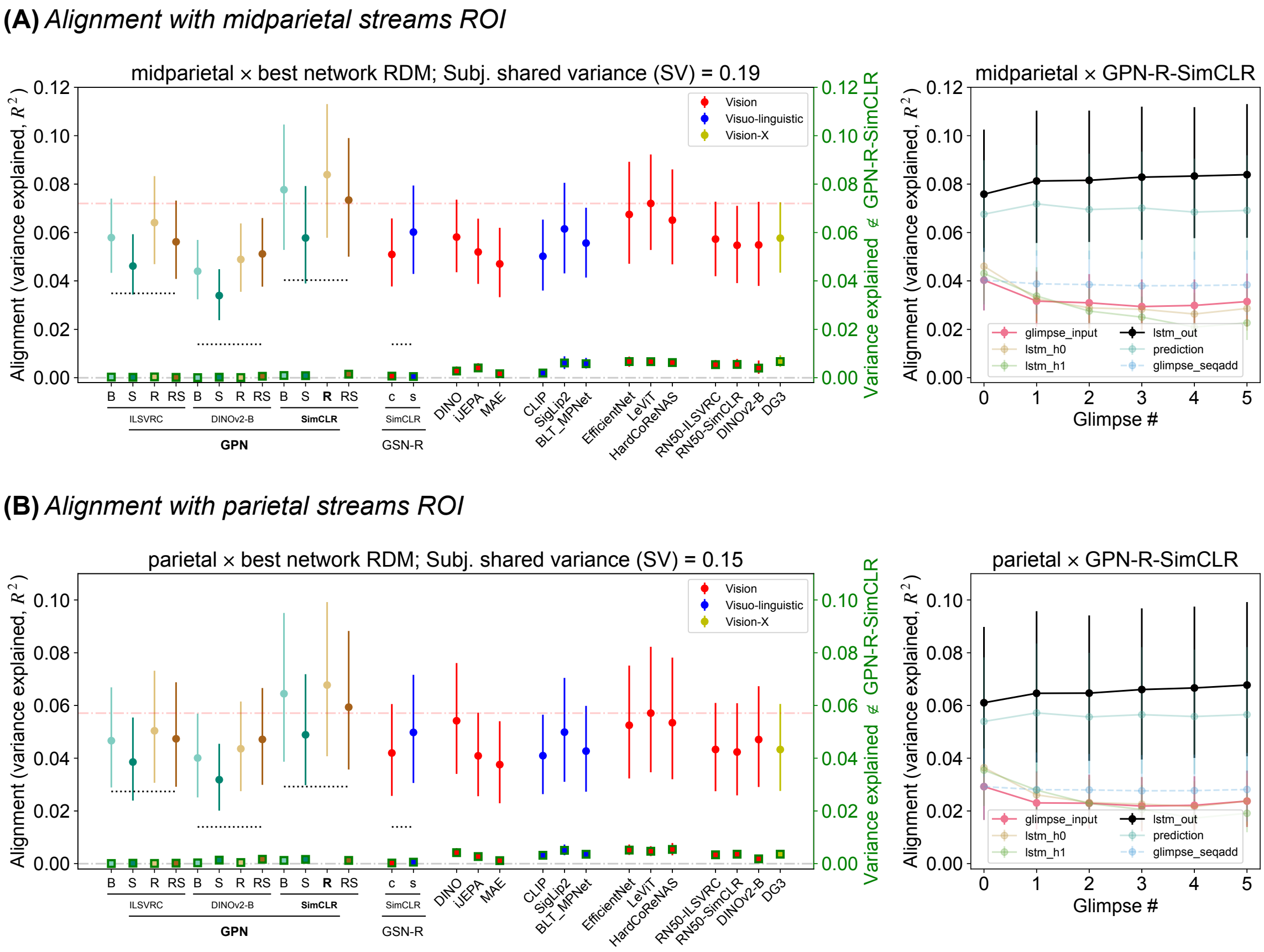

Figure S8: **Alignment of the GPN variants and comparison networks for the (A) midparietal, and (B) parietal streams ROIs** (see Fig. 3B for reference).

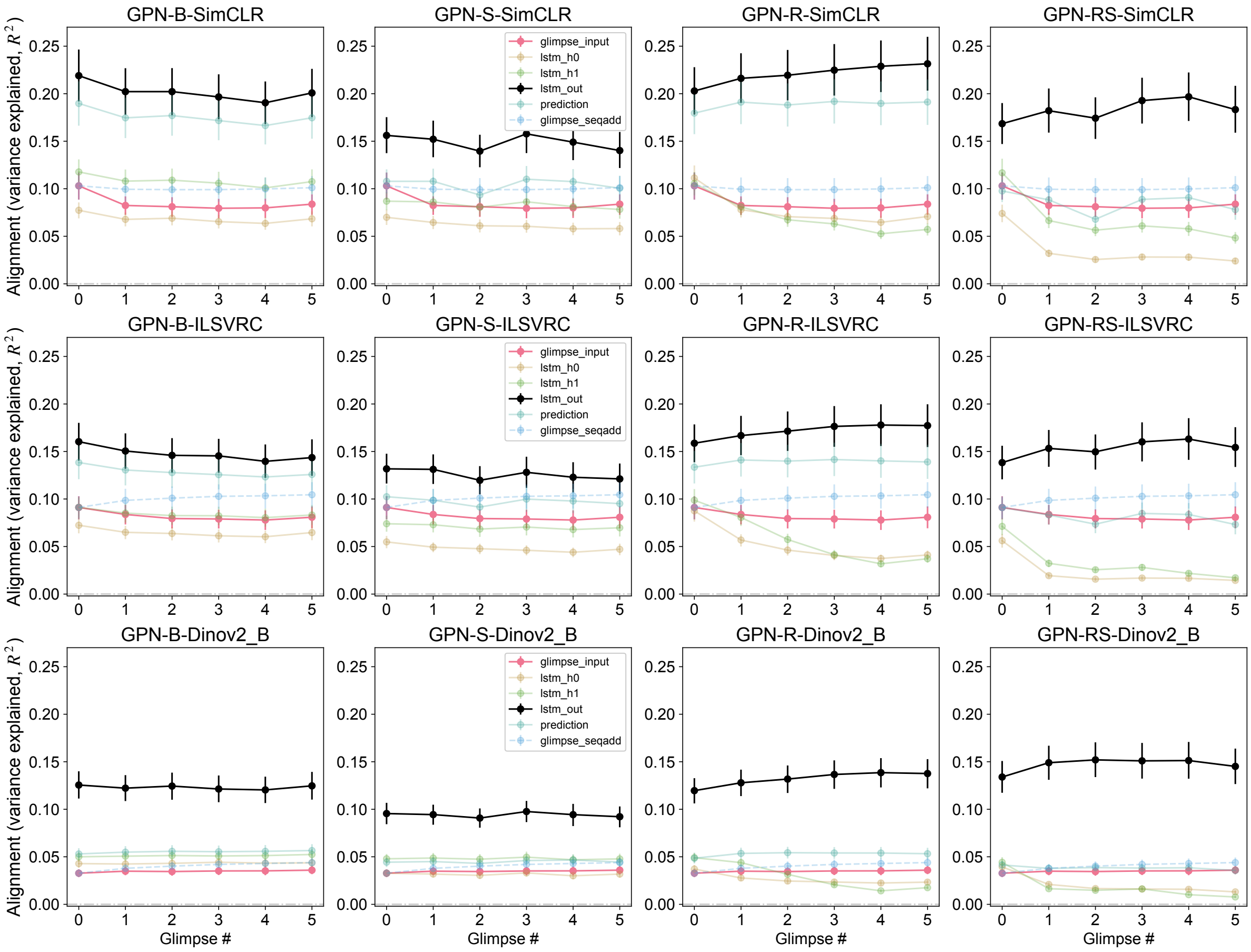

Figure S9: **VVC-alignment of GPN variants and backbones across layer and glimpse numbers in the sequences**. See Fig. 4B, for reference.

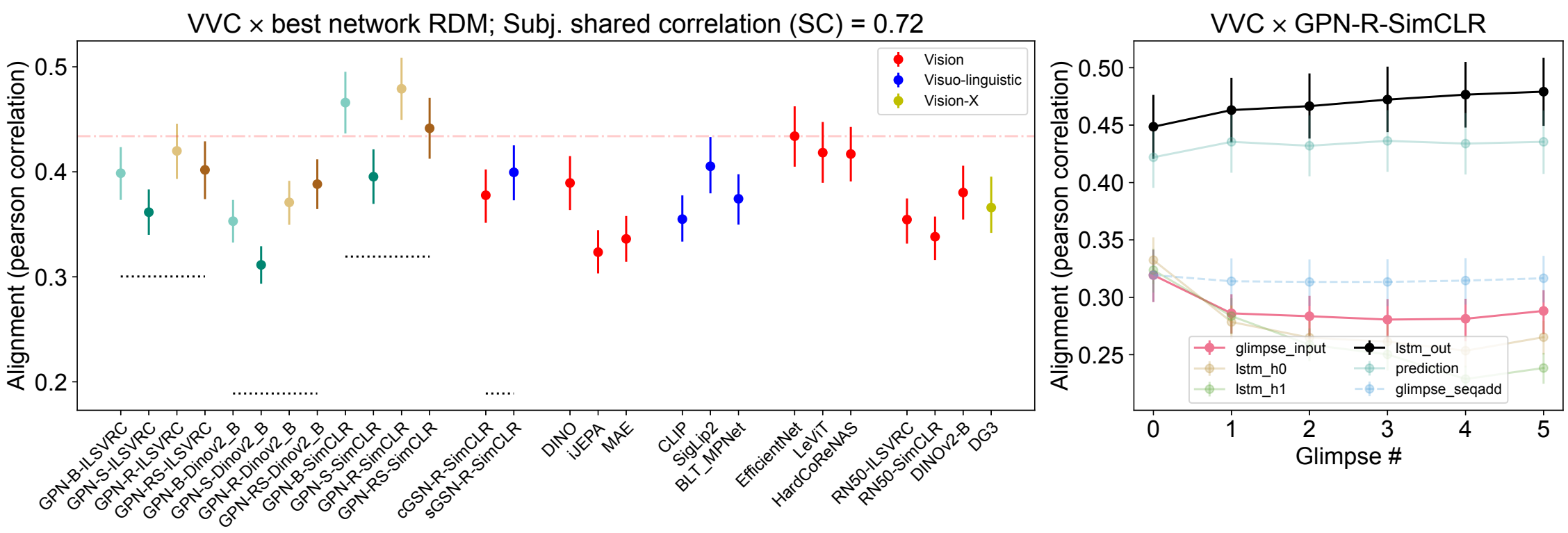

Figure S10: **VVC-alignment of GPNs and comparison networks, using Pearson correlation, instead of non-negative linear regression's R^2, as the measure** (see Fig. 3B for reference).

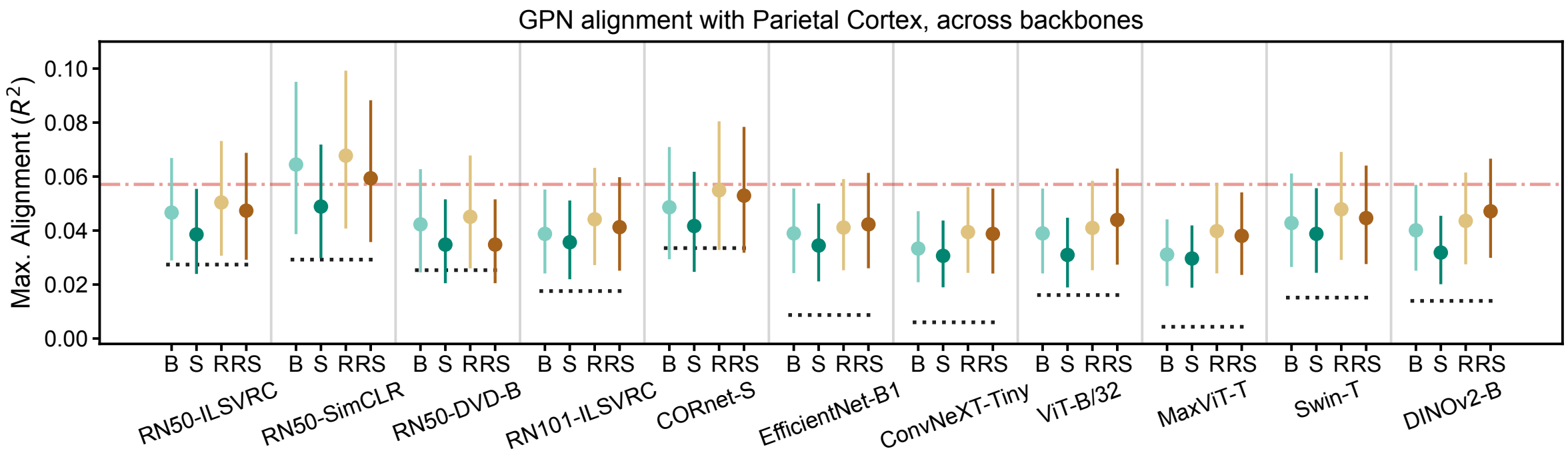

Figure S11: **Parietal Cortex-alignment of GPN variants trained over different backbones.** See Fig. S6, for methodological details.

Table S2: **GPNs and matched Glimpse Stitching Networks tested for VVC alignment.** For GPNs and GSNs, (d_{in}, d_{lstm}) reports the input and LSTM dropout values. The input/layer column reports the input and layer with the highest average VVC alignment.

Network	(d_{in}, d_{lstm})	Input / layer	R^2
GPNs			
GPN-B-ILSVRC	0.1, 0.1	glimpse 0, lstm_out	0.16
GPN-S-ILSVRC	0.1, 0.1	glimpse 0, lstm_out	0.13
GPN-R-ILSVRC	0.25, 0.25	glimpse 4, lstm_out	0.18
GPN-RS-ILSVRC	0.1, 0.1	glimpse 4, lstm_out	0.16
GPN-B-SimCLR	0.1, 0.1	glimpse 0, lstm_out	0.22
GPN-S-SimCLR	0.1, 0.1	glimpse 3, lstm_out	0.16
GPN-R-SimCLR	0.25, 0.1	glimpse 5, lstm_out	0.23
GPN-RS-SimCLR	0.1, 0.1	glimpse 4, lstm_out	0.20
GPN-B-Dinov2B	0.1, 0.1	glimpse 0, lstm_out	0.13
GPN-S-Dinov2B	0.1, 0.1	glimpse 3, lstm_out	0.10
GPN-R-Dinov2B	0.1, 0.1	glimpse 4, lstm_out	0.14
GPN-RS-Dinov2B	0.1, 0.1	glimpse 2, lstm_out	0.15
GSNs			
cGSN-R-SimCLR	0.5, 0.25	glimpse 0, joint_proj	0.14
sGSN-R-SimCLR	0.5, 0.5	glimpse 0, joint_proj	0.16

Table S3: **DINO-family self-supervised vision models tested for VVC alignment.** For each model, the table lists the source, the best-aligned input/layer, and the average VVC alignment. TH refers to PyTorch Hub; HF refers to Hugging Face.

Network	Source	Input / layer	R^2
dinov2_vitb14 (DINOv2-B)	TH: facebookresearch/dinov2	central glimpse, blocks.11.mlp.fc1	0.15
dinov2_vitl14	TH: facebookresearch/dinov2	central glimpse, blocks.23.norm2	0.15
dinov2_vitg14_reg	TH: facebookresearch/dinov2	central glimpse, blocks.22.norm2	0.10
dino_webssl_1b	HF: facebook/webssl-dino1b-full2b-224	central glimpse, encoder.layer.21.mlp.weights_in	0.12
dino_webssl_3b	HF: facebook/webssl-dino3b-full2b-224	central glimpse, encoder.layer.16.norm2	0.12
dino_webssl_7b	HF: facebook/webssl-dino7b-full8b-518	central glimpse, encoder.layer.20.norm2	0.11
dinov3_vitb16	HF: facebook/dinov3-vitb16-pretrain-lvd1689m	central glimpse, layer.8.norm2	0.13
dinov3_convnext_large	HF: facebook/dinov3-convnext-large-pretrain-lvd1689m	central glimpse, stages.2.layers.20.depthwise_conv	0.15
dinov3_vit7b16	HF: facebook/dinov3-vit7b16-pretrain-lvd1689m	central glimpse, layer.21.mlp.act_fn	0.12

Table S4: **iJEPA and MAE self-supervised vision models tested for VVC alignment.** For each model, the table lists the source, the best-aligned input/layer, and the average VVC alignment. HF refers to Hugging Face.

Network	Source	Input / layer	R^2
iJEPA			
ijepa_vith14_1k	HF: jmtzt/ijepa_vith14_1k	full scene, encoder.layer.24.intermediate.dense	0.10
ijepa_vitg16_22k	HF: jmtzt/ijepa_vitg16_22k	central glimpse, encoder.layer.31.intermediate.dense	0.11
MAE			
mae_vitb	HF: facebook/vit-mae-base	central glimpse, encoder.layer.8.intermediate.dense	0.08
mae_vitl	HF: facebook/vit-mae-large	central glimpse, encoder.layer.14.intermediate.dense	0.09
mae_vith	HF: facebook/vit-mae-huge	central glimpse, encoder.layer.15.intermediate.dense	0.11
mae_webssl_300m	HF: facebook/webssl-mae300m-full2b-224	full scene, encoder.layer.14.attention.attention	0.07
mae_webssl_1b	HF: facebook/webssl-mae1b-full2b-224	central glimpse, encoder.layer.21.intermediate.dense	0.10
mae_webssl_3b	HF: facebook/webssl-mae3b-full2b-224	central glimpse, encoder.layer.14.intermediate.dense	0.08

Table S5: **Vision-only supervised and architecture-optimized models tested for VVC alignment.** For each model, the table lists the source, the best-aligned input/layer, and the average VVC alignment.

Network	Source	Input / layer	R^2
EfficientNet			
efficientnet_b1	torchvision models	central glimpse, features.6.3.block.2.fc1	0.18
efficientnet_b3	torchvision models	central glimpse, features.6.4.block.2.fc1	0.19
efficientnet_b7	torchvision models	central glimpse, features.6.6.block.2.fc1	0.17
LeViT			
levit_128	timm: levit_128.fb_dist_in1k	central glimpse, stages.2.blocks.0.attn.qkv.linear	0.18
levit_256	timm: levit_256.fb_dist_in1k	full scene, stages.1.blocks.0.attn	0.13
levit_384	timm: levit_384.fb_dist_in1k	central glimpse, stages.1.blocks.0.attn	0.13
HardCoreNAS			
hardcorenas_a	timm: hardcorenas_a.miil_green_in1k	full scene, blocks.5.1.se.conv_reduce	0.17
hardcorenas_d	timm: hardcorenas_d.miil_green_in1k	central glimpse, blocks.5.1.se.conv_reduce	0.18
hardcorenas_f	timm: hardcorenas_f.miil_green_in1k	full scene, blocks.4.3.se.act1	0.17

Table S6: **Visual baseline models tested for VVC alignment.** For each model, the table lists the source, the best-aligned input/layer, and the average VVC alignment. DG3 denotes DeepGazeIII.

Network	Source	Input / layer	R^2
RN50-ILSVRC	torchvision models: v1	full scene, layer4.1.bn2	0.13
RN50-SimCLR	lightly-ai: simclrv1-imagenet1k-resnet50-1x	full scene, avgpool	0.12
DG3	github: matthias-k/DeepGaze	central glimpse, features.features.1.features.denseblock3.denselayer28.norm2	0.14

Table S7: **Vision-language and semantic baseline models tested for VVC alignment.** For each model, the table lists the source, the best-aligned input/layer, and the average VVC alignment. HF refers to Hugging Face.

Network	Source	Input / layer	R^2
CLIP			
clip_RN50	github: openai/CLIP: RN50	full scene, visual.layer4.1.bn1	0.11
clip_vitb	github: openai/CLIP: ViT-B/16	central glimpse, visual.transformer. resblocks.8.ln_2	0.13
clip_vitl	github: openai/CLIP: ViT-L/14@336px	central glimpse, visual.transformer. resblocks.11.attn	0.09
SigLIP			
siglip2_b	HF: google/siglip2-base-patch16-224	central glimpse, vision_model.encoder. layers.7.layer_norm2	0.17
siglip2_l	HF: google/siglip2-large-patch16-256	central glimpse, vision_model.encoder. layers.12.mlp.fc1	0.14
siglip2_g	HF: google/siglip2-giant-opt-patch16-384	central glimpse, vision_model.encoder. layers.15.mlp.fc1	0.13
Semantic baseline			
BLT_MPNet	Doerig et al (7)	full scene, layernorm_layer_9_time_0	0.13

REFERENCES

1. A. Doerig, R. P. Sommers, K. Seeliger, B. Richards, J. Ismael, G. W. Lindsay, K. P. Kording, T. Konkle, M. A. J. van Gerven, N. Kriegeskorte, T. C. Kietzmann, The neuroconnectionist research programme. *Nat. Rev. Neurosci.* **24**, 431–450 (2023).

2. B. A. Richards, T. P. Lillicrap, P. Beaudoin, Y. Bengio, R. Bogacz, A. Christensen, C. Clopath, R. P. Costa, A. de Berker, S. Ganguli, C. J. Gillon, D. Hafner, A. Kepecs, N. Kriegeskorte, P. Latham, G. W. Lindsay, K. D. Miller, R. Naud, C. C. Pack, P. Poirazi, P. Roelfsema, J. Sacramento, A. Saxe, B. Scellier, A. C. Schapiro, W. Senn, G. Wayne, D. Yamins, F. Zenke, J. Zylberberg, D. Therien, K. P. Kording, A deep learning framework for neuroscience. *Nat. Neurosci.* **22**, 1761–1770 (2019).

3. D. L. K. Yamins, J. J. DiCarlo, Using goal-driven deep learning models to understand sensory cortex. *Nat. Neurosci.* **19**, 356–365 (2016).

4. M. V. Peelen, P. E. Downing, Category selectivity in human visual cortex: Beyond visual object recognition. *Neuropsychologia* **105**, 177–183 (2017).

5. R. A. Epstein, C. I. Baker, Scene perception in the human brain. *Annu. Rev. Vis. Sci.* **5**, 373–397 (2019).

6. A. Y. Wang, K. Kay, T. Naselaris, M. J. Tarr, L. Wehbe, Better models of human high-level visual cortex emerge from natural language supervision with a large and diverse dataset. *Nat. Mach. Intell.* **5**, 1415–1426 (2023).

7. A. Doerig, T. C. Kietzmann, E. Allen, Y. Wu, T. Naselaris, K. Kay, I. Charest, High-level visual representations in the human brain are aligned with large language models. *Nat. Mach. Intell.* **7**, 1220–1234 (2025).

8. M. C. Frank, Bridging the data gap between children and large language models. *Trends Cogn. Sci.* **27**, 990–992 (2023).

9. C. Conwell, E. McMahon, A. Jagadeesh, K. Vinken, S. Sharma, J. S. Prince, G. A. Alvarez, T. Konkle, M. Livingstone, L. Isik, Monkey see, model knew: Large language models accurately predict visual brain responses in humans and non-human primates. bioRxiv 641284 [Preprint] (2025). https://doi.org/10.1101/2025.03.05.641284.

10. A. L. Yarbus, "Eye movements during perception of complex objects," in *Eye Movements and Vision*, A. L. Yarbus, Ed. (Springer, 1967), pp. 171–211.

11. D. Kaiser, G. L. Quek, R. M. Cichy, M. V. Peelen, Object vision in a structured world. *Trends Cogn. Sci.* **23**, 672–685 (2019).

12. C. Summerfield, F. Luyckx, H. Sheahan, Structure learning and the posterior parietal cortex. *Prog. Neurobiol.* **184**, 101717 (2020).

13. J. L. Elman, Finding structure in time. *Cognit. Sci.* **14**, 179–211 (1990).

14. A. vanden Oord, Y. Li, O. Vinyals, Representation learning with contrastive predictive coding. arXiv:1807.03748 [cs.LG] (2019).

15. S. Bakhtiari, P. Mineault, T. Lillicrap, C. Pack, B. Richards, "The functional specialization of visual cortex emerges from training parallel pathways with self-supervised predictive learning," in *Advances in Neural Information Processing Systems* (Curran Associates Inc., 2021), vol. 34, pp. 25164–25178.

16. M. Kümmerer, M. Bethge, T. S. A. Wallis, DeepGaze III: Modeling free-viewing human scanpaths with deep learning. *J. Vis.* **22**, 7 (2022).

17. T.-Y. Lin, M. Maire, S. Belongie, J. Hays, P. Perona, D. Ramanan, P. Dollár, C. L. Zitnick, "Microsoft COCO: Common Objects in Context," in *Computer Vision - ECCV 2014*, D. Fleet, T. Pajdla, B. Schiele, T. Tuytelaars, Eds. (Springer International Publishing, 2014), pp. 740–755.

18. T. B. Brown, B. Mann, N. Ryder, M. Subbiah, J. Kaplan, P. Dhariwal, A. Neelakantan, P. Shyam, G. Sastry, A. Askell, S. Agarwal, A. Herbert-Voss, G. Krueger, T. Henighan, R. Child, A. Ramesh, D. M. Ziegler, J. Wu, C. Winter, C. Hesse, M. Chen, E. Sigler, M. Litwin, S. Gray,

B. Chess, J. Clark, C. Berner, S. M. Candlish, A. Radford, I. Sutskever, D. Amodei, "Language models are few-shot learners," in *Advances in Neural Information Processing Systems* (Curran Associates Inc., 2020), vol. 33, pp. 1877–1901.

19. E. J. Allen, G. St-Yves, Y. Wu, J. L. Breedlove, J. S. Prince, L. T. Dowdle, M. Nau, B. Caron, F. Pestilli, I. Charest, J. B. Hutchinson, T. Naselaris, K. Kay, A massive 7T fMRI dataset to bridge cognitive neuroscience and artificial intelligence. *Nat. Neurosci.* **25**, 116–126 (2022).

20. N. Kriegeskorte, M. Mur, P. A. Bandettini, Representational similarity analysis—Connecting the branches of systems neuroscience. *Front. Syst. Neurosci.* **2**, 4 (2008).

21. R. Epstein, N. Kanwisher, A cortical representation of the local visual environment. *Nature* **392**, 598–601 (1998).

22. M. Caron, H. Touvron, I. Misra, H. Jégou, J. Mairal, P. Bojanowski, A. Joulin, "Emerging Properties in Self-Supervised Vision Transformers," in *Proceedings of the IEEE/CVF International Conference on Computer Vision* (IEEE, 2021), pp. 9650–9660.

23. M. Assran, Q. Duval, I. Misra, P. Bojanowski, P. Vincent, M. Rabbat, Y. L. Cun, N. Ballas, "Self-supervised learning from images with a joint-embedding predictive architecture," in *Proceedings of the IEEE/CVF Conference on Computer Vision and Pattern Recognition* (IEEE, 2023), pp. 15619–15629.

24. K. He, X. Chen, S. Xie, Y. Li, P. Dollár, R. Girshick, "Masked autoencoders are scalable vision learners," in *Proceedings of the IEEE/CVF Conference on Computer Vision and Pattern Recognition* (IEEE, 2022), pp. 16000–16009.

25. A. Radford, J. W. Kim, C. Hallacy, A. Ramesh, G. Goh, S. Agarwal, G. Sastry, A. Askell, P. Mishkin, J. Clark, G. Krueger, I. Sutskever, "Learning Transferable Visual Models From Natural Language Supervision," in *Proceedings of the 38th International Conference on Machine Learning (PMLR),* vol. 139 of *Proceedings of Machine Learning Research* (PLMR, 2021), pp. 8748–8763.

26. M. Tschannen, A. Gritsenko, X. Wang, M. F. Naeem, I. Alabdulmohsin, N. Parthasarathy, T. Evans, L. Beyer, Y. Xia, B. Mustafa, O. Hénaff, J. Harmsen, A. Steiner, X. Zhai, "SigLIP 2: Multilingual vision-language encoders with improved semantic understanding, localization, and dense features. arXiv:2502.14786 [cs.CV] (2025).

27. C. Conwell, J. S. Prince, K. N. Kay, G. A. Alvarez, T. Konkle, A large-scale examination of inductive biases shaping high-level visual representation in brains and machines. *Nat. Commun.* **15**, 9383 (2024).

28. M. Tan, Q. V. Le, "EfficientNet: Rethinking model scaling for convolutional neural networks," in *Proceedings of the 36th International Conference on Machine Learning (PMLR)*, vol. 97 of *Proceedings of Machine Learning Research* (PMLR, 2019), pp. 6105–6114.

29. B. Graham, A. El-Nouby, H. Touvron, P. Stock, A. Joulin, H. Jégou, M. Douze, "LeViT: A vision transformer in ConvNet's clothing for faster inference," in *Proceedings of the IEEE/CVF International Conference on Computer Vision* (IEEE, 2021), pp. 12259–12269.

30. N. Nayman, Y. Aflalo, A. Noy, L. Zelnik, "HardCoRe-NAS: Hard constrained differentiable neural architecture search," in *Proceedings of the 38th International Conference on Machine Learning* (PMLR, 2021), pp. 7979–7990.

31. M. Huh, B. Cheung, T. Wang, P. Isola, "Position: The platonic representation hypothesis," in *Proceedings of the 41st International Conference on Machine Learning* (PMLR, 2024), pp. 20617–20642.

32. Z. S. Harris, Distributional structure. *Word* **10**, 146–162 (1954).

33. T. Mikolov, K. Chen, G. Corrado, J. Dean, Efficient estimation of word representations in vector space. arXiv:1301.3781 [cs.CL] (2013).

34. M. Bar, E. Aminoff, Cortical analysis of visual context. *Neuron* **38**, 347–358 (2003).

35. M. F. Bonner, R. A. Epstein, Object representations in the human brain reflect the co-occurrence statistics of vision and language. *Nat. Commun.* **12**, 4081 (2021).

36. D. Kaiser, M. V. Peelen, Transformation from independent to integrative coding of multi-object arrangements in human visual cortex. *Neuroimage* **169**, 334–341 (2018).

37. M. V. Chafee, B. B. Averbeck, D. A. Crowe, Representing spatial relationships in posterior parietal cortex: Single neurons code object-referenced position. *Cereb. Cortex* **17**, 2914–2932 (2007).

38. V. Ayzenberg, M. Behrmann, The dorsal visual pathway represents object-centered spatial relations for object Recognition. *J. Neurosci.* **42**, 4693–4710 (2022).

39. J. K. O'Regan, A. Noë, A sensorimotor account of vision and visual consciousness. *Behav. Brain Sci.* **24**, 939–973 (2001).

40. Y. LeCun, A path towards autonomous machine intelligence version 0.9.2, 2022-06-27. *OpenReview* **62**, 1–62 (2022).

41. R. P. N. Rao, D. C. Gklezakos, V. Sathish, Active predictive coding: A unifying neural model for active perception, compositional learning, and hierarchical planning. *Neural Comput.* **36**, 1–32 (2023).

42. D. Linsley, P. Feng, T. Serre, Better artificial intelligence does not mean better models of biology. *Trends Cogn. Sci.* **30**, 599–610 (2026).

43. A. Paszke, S. Gross, F. Massa, A. Lerer, J. Bradbury, G. Chanan, T. Killeen, Z. Lin, N. Gimelshein, L. Antiga, A. Desmaison, A. Köpf, E. Yang, Z. De Vito, M. Raison, A. Tejani, S. Chilamkurthy, B. Steiner, L. Fang, J. Bai, S. Chintala, PyTorch: An imperative style, high-performance deep learning library. arXiv:1912.01703 [cs.LG] (2019).

44. J. A. F. Thompson, H. Sheahan, T. Dumbalska, J. D. Sandbrink, M. Piazza, C. Summerfield, Zero-shot counting with a dual-stream neural network model. *Neuron* **112**, 4147–4158.e5 (2024).

45. R. Legras, A. Gaudric, K. Woog, Distribution of cone density, spacing and arrangement in adult healthy retinas with adaptive optics flood illumination. *PLOS ONE* **13**, e0191141 (2018).

46. K. He, X. Zhang, S. Ren, J. Sun, "Deep residual learning for image recognition," in *Proceedings of the IEEE Conference on Computer Vision and Pattern Recognition* (IEEE, 2016), pp. 770–778.

47. T. Chen, S. Kornblith, M. Norouzi, G. Hinton, A simple framework for contrastive learning of visual representations. arXiv:2002.05709 [cs.LG] (2020).

48. M. Oquab, T. Darcet, T. Moutakanni, H. Vo, M. Szafraniec, V. Khalidov, P. Fernandez, D. Haziza, F. Massa, A. El-Nouby, M. Assran, N. Ballas, W. Galuba, R. Howes, P.-Y. Huang, S.-W. Li, I. Misra, M. Rabbat, V. Sharma, G. Synnaeve, H. Xu, H. Jegou, J. Mairal, P. Labatut, A. Joulin, P. Bojanowski, DINOv2: Learning robust visual features without supervision. *Trans. Mach. Learn. Res.* (2024). https://openreview.net/forum?id=a68SUt6zFt.

49. R. Balestriero, Y. LeCun, Contrastive and non-contrastive self-supervised learning recover global and local spectral embedding methods. *Adv. Neural Inf. Proces. Syst.* **35**, 26671–26685 (2022).

50. R. Balestriero, Y. LeCun, Learning by reconstruction produces uninformative features for perception. arXiv:2402.11337 [cs.CV] (2024).

51. F. Pedregosa, G. Varoquaux, A. Gramfort, V. Michel, B. Thirion, O. Grisel, M. Blondel, A. Müller, J. Nothman, G. Louppe, P. Prettenhofer, R. Weiss, V. Dubourg, J. Vanderplas, A. Passos, D. Cournapeau, M. Brucher, M. Perrot, É. Duchesnay, Scikit-learn: Machine learning in Python. *J. Mach. Learn. Res.* **12**, 2825–2830 (2011).

52. A. J. Conger, A revised definition for suppressor variables: A guide to their identification and interpretation. *Educ. Psychol. Meas.* **34**, 35–46 (1974).

53. P. Virtanen, R. Gommers, T. E. Oliphant, M. Haberland, T. Reddy, D. Cournapeau, E. Burovski, P. Peterson, W. Weckesser, J. Bright, Stéfan J van der Walt, M. Brett, J. Wilson, K Jarrod Millman, N. Mayorov, A. R. J. Nelson, E. Jones, R. Kern, E. Larson, C J Carey, İ. Polat, Y. Feng, E. W. Moore, J. V. Plas, D. Laxalde, J. Perktold, R. Cimrman, I. Henriksen, E A Quintero, C. R. Harris, A. M. Archibald, A. H. Ribeiro, F. Pedregosa, P. van Mulbregt, SciPy 1.0

Contributors, SciPy 1.0: Fundamental algorithms for scientific computing in Python. *Nat. Methods* **17**, 261–272 (2020).

54. S. Seabold, J. Perktold, “Statsmodels: Econometric and Statistical Modeling with Python,” in *Proceedings of the 9th Python in Science Conference* (SciPy, 2010), pp. 92–96.

55. P. Smolensky, Tensor product variable binding and the representation of symbolic structures in connectionist systems. *Artif Intell* **46**, 159–216 (1990).

56. Z. Lu, S. Thorat, R. M. Cichy, T. C. Kietzmann, Adopting a human developmental visual diet yields robust and shape-based AI vision. *Nat. Mach. Intell.* **8**, 735–748 (2026).

57. J. Kubilius, M. Schrimpf, A. Nayebi, D. Bear, D. L. K. Yamins, J. J. DiCarlo, CORnet: Modeling the neural mechanisms of core object recognition. bioRxiv 408385 [Preprint] (2018). https://doi.org/10.1101/408385.

58. Z. Liu, H. Mao, C.-Y. Wu, C. Feichtenhofer, T. Darrell, S. Xie, “A ConvNet for the 2020s,” in *Proceedings of the IEEE/CVF conference on computer vision and pattern recognition* (IEEE, 2022), pp. 11976–11986.

59. A. Dosovitskiy, L. Beyer, A. Kolesnikov, D. Weissenborn, X. Zhai, T. Unterthiner, M. Dehghani, M. Minderer, G. Heigold, S. Gelly, J. Uszkoreit, N. Houlsby, “An image is worth 16x16 words: Transformers for image recognition at scale,” in *International Conference on Learning Representations* (OpenReview.net, 2021).

60. Z. Tu, H. Talebi, H. Zhang, F. Yang, P. Milanfar, A. Bovik, Y. Li, “Maxvit: Multi-axis vision transformer,” in *European Conference on Computer Vision*, vol. 13684 of *Leecture Notes in Computer Science* (Springer, 2022), pp. 459–479.

61. Z. Liu, Y. Lin, Y. Cao, H. Hu, Y. Wei, Z. Zhang, S. Lin, B. Guo, “Swin transformer: Hierarchical vision transformer using shifted windows,” in *Proceedings of the IEEE/CVF international conference on computer vision* (IEEE, 2021), pp. 10012–10022.
